# Cysteine protease cathepsin B promotes lysosome integrity to extend the lifespan of alternative day fasting worms

**DOI:** 10.1111/acel.14286

**Published:** 2024-07-24

**Authors:** Xue Yin, Fangzhou Dai, Dongyang Ran, Yutong Zhang, Zhi Qu, Shanqing Zheng

**Affiliations:** ^1^ School of Basic Medical Sciences Henan University Kaifeng China; ^2^ School of Nursing and Health Henan University Kaifeng China; ^3^ Laboratory of Cell Signal Transduction, Henan Provincial Engineering Centre for Tumor Molecular Medicine Medical School of Henan University Kaifeng China; ^4^ The Zhongzhou Laboratory for Integrative Biology Zhengzhou Henan China

**Keywords:** alternative day fasting, *C. elegans*, cysteine protease cathepsin B, lifespan, lysosome

## Abstract

Alternative day fasting (ADF) has been shown to enhance the lifespan of animals. However, human trials evaluating the efficacy of ADF have only recently emerged, presenting challenges due to the extreme nature of this dietary regimen. To better understand the effects of ADF, we investigated its impact using *Caenorhabditis elegan*s as a model organism. Our findings reveal that ADF extends the lifespan of worms nourished on animal‐based protein source, while those fed with plant‐based protein as the primary protein source do not experience such benefits. Remarkably, initiating ADF during midlife is sufficient to prolong lifespan, whereas implementation during youth results in developmental damage, and in older age, fails to provide additional extension effects. Furthermore, we discovered that midlife ADF up‐regulates the expression of two cysteine protease cathepsin B genes, *cpr‐2* and *cpr‐5*, which preserve lysosomal integrity and enhance its function in digesting aggregated proteins, as well as enhancing lipid metabolism and ameliorating neurodegenerative disease markers and phenomena during aging. This suggests that midlife ADF has long lasting anti‐aging effects and may delay the onset of related diseases, specifically in animals consuming animal‐based protein source. These findings offer valuable insights into the effects of ADF and provide guidance for future research and potential applications in individuals.

## Graphical abstract

Proposed model of how alternative day fasting (ADF) affects the lifespan of worms cultured on animal‐based protein source. ADF on young worms shortens lifespan and causes embryonic developmental issues, potentially due to improper wingless/integrated (WNT) and transforming growth factor‐β (TGF‐β) pathway activation. In contrast, ADF on midlife worms significantly extends lifespan by inducing the expression of *cpr‐2* and *cpr‐5*, enhancing lysosomal integrity via cathepsin B. This involves longevity pathways like DAF‐16, SKN‐1, and EAT‐2, and reduces fat content, possibly involving HLH‐30‐regulated lipophagy. Midlife ADF also helps digest protein aggregates in later life. No effects were observed with ADF on old worms.

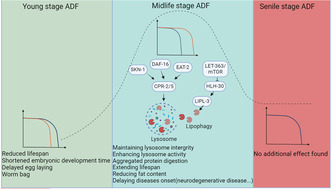

**Abbreviations**
ADF: alternative day fasting
CR: calorie restriction
DR: dietary restriction
E.coli OP50: escherichia coli OP50
EDTA: ethylenediaminetetraacetic acid
GFP: green fluorescent protein
GO: gene ontology
IF: intermittent fasting
IIS: insulin/IGF‐1 signaling
KEGG: kyoto encyclopedia of genes and genomes
L1: larval stage 1
L2: larval stage 2
L3: larval stage 3
L4: larval stage 4
LSG: LysoSensor Green DND‐189
LTR: LysoTracker Red
mTOR: mammalian target of rapamycin
NGM: nematode growth medium
PMSF: phenylmethanesulfonyl fluoride
qRT‐PCR: quantitative real‐time PCR
RNAi: RNA interference
TGF‐β: transforming growth factor‐β
WNT: wingless/integrated
YFP: yellow fluorescent protein
α‐syn: alpha‐synuclein

## 1 INTRODUCTION

Dietary restriction (DR) has emerged as a promising strategy for improving health‐span, preventing age‐related diseases (Longo & Anderson, 2022), and delaying their onset in humans (Green et al., 2021). Among the various DR regimens, including calorie restriction (CR) and intermittent fasting (IF), both have shown efficacy in increasing lifespan and enhancing disease resistance across animals and humans (Flanagan et al., 2020). IF, characterized by intermittent food intake during feeding, offers a viable alternative to CR for improving lifespan through different underlying mechanisms (Deota et al., 2023; Waziry et al., 2023). Various IF regimens have garnered attention. Of particular interest is alternative day fasting (ADF), an extreme form of CR that robustly extends the lifespan of model organisms like *C. elegans* (Honjoh et al., 2009). However, the diverse forms of IF across different cohorts complicate interpretation, and the precise physiological and molecular mechanisms remain unclear.

The beneficial effects of DR, dependent on fasting timing and specific macronutrient reduction, have been observed (Longo et al., 2021; Solon‐Biet et al., 2014). Although human trials investigating the efficacy of ADF have only recently emerged, long‐term adherence to ADF and controlling specific dietary components during fasting present significant challenges for both mammals and human trials. To understanding the most effective stage of lifespan to implement ADF is essential, as is recognizing the critical role of protein restriction and source in extending lifespan (Solon‐Biet et al., 2015). However, challenges arise in utilizing protein sources in diets for studying underlying mechanisms, and little is known about how dietary modifications in protein source affect the molecular pathways governing aging. To further address the effects of timing of ADF and the food ingredients on health, in this study, we provided *C. elegans* with different protein sources and implemented ADF at young, midlife, and old ages to explore its effects and mechanisms on lifespan.

Exploring the link between lysosomal function and health‐span/lifespan during food deprivation presents a promising avenue for investigation. Genetic overexpression studies in various organisms suggest a positive correlation between lysosomal function and longevity. Lysosomes, traditionally regarded as cellular recycling centers, exhibit variability in number, composition, and function in response to environmental cues and cellular needs. Age‐related changes in lysosomes, including alterations in size, number, content, and hydrolase activity, underscore their pivotal role in cellular aging (Carmona‐Gutierrez et al., 2016). However, the relationship between lysosomal integrity, lysosomal proteases, and ADF remains largely unexplored.

Increased lysosomal gene expression with age is believed to be a compensatory response to changes in protein homeostasis (Sun et al., 2020). For instance, overexpression of the TFEB homolog HLH‐30, a regulator of lysosome biogenesis in *C. elegans*, has been associated with longevity, potentially through autophagy induction (Lapierre et al., 2013; O'Rourke & Ruvkun, 2013). Similarly, the overexpression of lysosomal acid lipase *lipl‐4* in *C. elegans* correlates with extended lifespan (Lapierre et al., 2014). Lysosomes, comprising various soluble acid hydrolases and membrane proteins, play crucial roles in cellular homeostasis, autophagy, and apoptosis (Honey & Rudensky, 2003). Cathepsins, major lysosomal cysteine proteases, have specific functions tied to tissue localization and lysosomal integrity (Zhang et al., 2023). Overexpression of *Pep4*, a lysosomal cathepsin D homolog, has been shown to extend lifespan independently of *S. cerevisiae* (Carmona‐Gutiérrez et al., 2011). However, the relationship between lysosomal integrity, lysosomal proteases, and ADF remains largely unexplored.

In this work, our study demonstrated that implementing ADF exclusively on midlife stage worms cultured on animal‐based protein sources significantly extends lifespan. This extension is facilitated by the up‐regulation of proteases belonging to the Cathepsin B family, specifically coded by *cpr‐2* and *cpr‐5*. In contrast, ADF administered to worms cultured on plant‐based protein sources did not result in lifespan extension. Our study highlights the efficacy of ADF in midlife‐aged worms, where it triggers the expression of cathepsin genes, thereby preserving lysosomal activity and supporting total lifespan. These findings suggest potential benefits for neurodegenerative diseases and emphasize the importance of considering both the timing and dietary composition when implementing ADF.

## 2 RESULTS

### 2.1 Midlife age ADF promote lifespan of *C. elegans* cultured on animal‐based protein source

**FIGURE 1:**
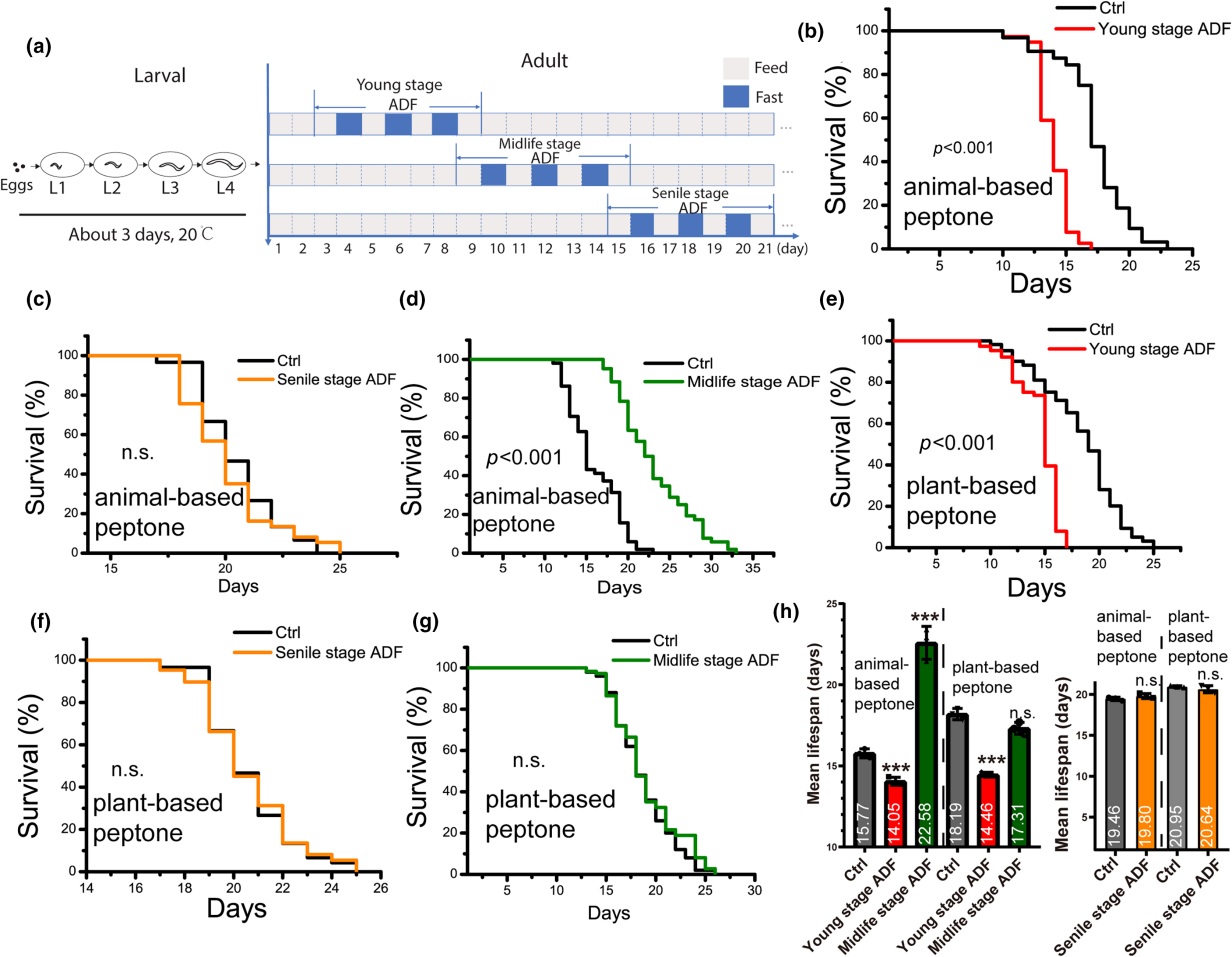
ADF performed on midlife‐aged worms cultured on animal‐based protein source extends lifespan. (a) Schematic representation of ADF performed on worms of different ages. (b–d) The lifespans of worms cultured on animal‐based peptone when subjected to ADF. (e–g) The lifespans of worms cultured on plant‐based peptone when subjected to ADF. (h) Summary of mean lifespans. Data show mean lifespan ± SEM. Wildtype N2 worms were used in all lifespan tests, cultured at 20°C using standard methods. Each survival curve represents at least three independent repeats. *p*‐Values were determined using the log‐rank test. ADF, alternative day fasting; Ctrl, control worms; n.s., no significant difference. ***p < 0.001. The detailed data of lifespan was summarized in Table S1 in supporting information.

Extreme and long‐term ADF is more easily conducted on animal models such as *C. elegans*. To provide more precise recommendations on ADF for individuals of different ages, we implemented an ADF regimen at different life stages: young, midlife, and senile age of *C. elegans* (Figure 1a). We observed that ADF applied to young animals resulted in a shorter lifespan (Figure 1b,h), while ADF applied to senile animals did not alter overall lifespan (Figure 1c,h). Interestingly, ADF applied to midlife age worms significantly improved their lifespan (Figure 1d,h). These results suggest that ADF applied only to midlife age animals is sufficient to support lifespan extension. On feasting days, individuals typically have no restrictions on the types or quantities of foods consumed. Considering that *C. elegans* were cultured on NGM plates and provided *E. coli* OP50 as standard food, the worms also intake a variety of nutrients to support their development from the NGM media (Zečić et al., 2019). Peptone is a crucial proteinaceous factor for continuous growth and reproduction. To test whether the protein source affects the effects of ADF on lifespan extension, we replaced animal‐based peptone with soy peptone in the NGM plates. Interestingly, we found that even young (Figure 1e,h) and senile (Figure 1f,h) worms showed similar effects on lifespan with ADF intervention, whereas midlife age worms undergoing ADF lost the lifespan extension (Figure 1g,h). These results suggest that while ADF can cause damage to young life stage animals regardless of the protein source media, the ADF lifespan extension strategy is only applicable to midlife age animals cultured on animal‐based protein source media.

### 2.2 Early ADF causes serious developmental damage

We observed that ADF in young stage worms led to the production of premature eggs, reducing embryonic developmental time (Figure S1a). However, the timing of egg laying was significantly delayed (Figure S1b), resulting in eggs being hatched inside the worms (Figure S1c). These results indicate serious embryonic and post‐embryonic damage caused by ADF. To avoid internal hatching affecting the lifespan, we used the sterile strain *fem‐3*(*e2006*) to retest the lifespan of young stage worms performed to ADF. Our results further confirmed that young stage ADF reduced the lifespan of worms cultured on plant‐ or animal‐based peptone (Table S1). To understand how ADF affects young adult stage worms, we conducted RNA‐seq analysis. Results revealed significant changes in genes related to development and egg laying pathways, including transforming growth factor‐β (TGF‐β) and wingless/integrated (WNT) (Figure S1d). Two genes in these pathways, *cyd‐1* and *cfz‐2*, were significantly down‐regulated by ADF (Figure S1e), which was confirmed by real‐time PCR (Figure S1f). Further tests on lifespan using *cyd‐1* and *cfz‐2* mutants showed that ADF did not further reduce lifespan (Figure S1g–i), suggesting that ADF‐induced embryonic development damage may be related to improper activation of WNT and TGF‐β pathways. These results suggested ADF applied to young‐stage worms led to reduced lifespan and developmental deficiencies, including eggs hatching inside the worms, potentially due to disrupted developmental signals.

### 2.3 Midlife age ADF promotes lifespan through *cpr‐2* and *cpr‐5*

**FIGURE 2:**
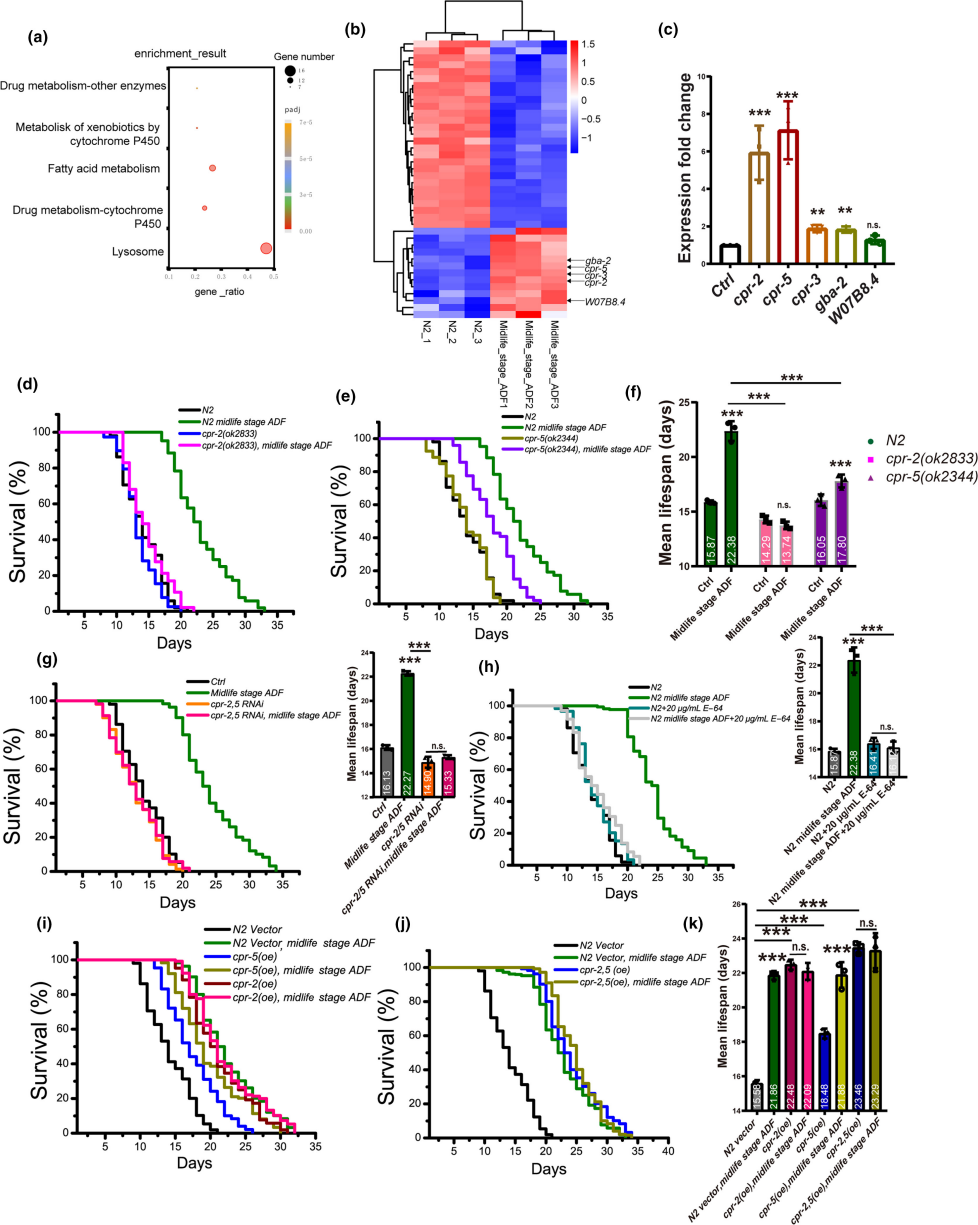
Midlife stage alternative day fasting (ADF) promotes lifespan through lysosomal proteases of the Cathepsin B family coding genes: *Cpr‐2* and *cpr‐5*. (a) RNA‐seq results suggest that lysosome activity is enriched in ADF midlife age worms. (b) Significantly changed genes in ADF‐treated midlife age worms. Heatmap: Genes significantly changed (>1.5 fold). (c) Changes in lysosomal protease genes in ADF midlife age worms were verified using real‐time PCR. **p < 0.01; ***p < 0.001, n.s., no significant difference. *t*‐test. Ctrl, Control housekeeping gene *cdc‐42*. (d) *cpr‐2* and (e) *cpr‐5* mutants significantly reduced the lifespan extension of ADF performed on midlife age worms. (f) Summary of mean lifespans. Ctrl: Worms without ADF. (g) *cpr‐2*, *cpr‐5* double RNAi abolished the lifespan extension of ADF performed on midlife age worms. Ctrl: Control, worms fed with RNAi L4440 strain. Summary of mean lifespans. (h) E‐64, the inhibitor of protease, abolished the lifespan extension of ADF performed on midlife age worms. Summary of mean lifespans. (i) Overexpression of *cpr‐2*, *cpr‐5*, or both (j) can extend the lifespan of worms; ADF performed on these worms failed to further extend lifespan. (k) Summary of mean lifespans of transgenic worms. (i–k) Vector: Empty expression control vector L2528. Each survival curve is representative of at least three independent repeats. *p*‐Values were determined using the log‐rank test. ***p < 0.001, n.s., no significant difference. The detailed data of lifespan was summarized in Table S2 in supporting information.

As midlife age ADF is sufficient to extend the longevity of worms, we aim to understand how ADF promotes the survival and health of midlife age worms. Subsequently, we conducted a genome‐wide analysis using RNA‐seq to compare the gene expression profiles of midlife age worms treated with ADF. The RNA‐seq data revealed a significant activation of lysosome function by ADF (Figure 2a). Focusing on lysosome function, we identified several genes upregulated by ADF more than twofold with statistical significance (Figure 2b). Real‐time PCR further confirmed the upregulation of *cpr‐2* and *cpr‐5* in midlife stage worms by ADF, with their expression increasing more than fivefold (Figure 2c), suggesting their involvement in ADF‐induced lifespan extension. To assess the role of these genes in lifespan extension induced by ADF, we conducted lifespan experiments on midlife stage worms with mutations in *cpr‐2* (Figure 2d,f) and *cpr‐5* (Figure 2e,f). The *cpr‐2* mutants almost completely abolished the extension effect of ADF. Knocking down both *cpr‐2* and *cpr‐5* nearly abolished the lifespan extension function of ADF (Figure 2g). Since *cpr‐2* and *cpr‐5* genes encode proteins with significant similarity to proteases of the Cathepsin B family, and enable cysteine‐type endopeptidase activity, we used E‐64, a cysteine protease inhibitor, to treat midlife stage worms undergoing ADF. Results showed that E‐64 blocked the lifespan extension effects of ADF (Figure 2h), confirming that ADF extends the lifespan of midlife age worms through *cpr‐2* and *cpr‐5*. Additionally, we overexpressed *cpr‐2* and *cpr‐5*, respectively, and found that they extended the lifespan of worms (Figure 2i,k), with *cpr‐2* overexpression showing a stronger extension effect. Furthermore, ADF‐treated midlife stage worms did not further extend the lifespan of *cpr‐2/5* overexpressed worms (Figure 2j,k). These results suggest that the lifespan extension by ADF in midlife age worms mainly occurs through activating *cpr‐2/5* regulated lysosome activity. We also performed tissue‐specific knockdowns of *cpr‐2* and *cpr‐5*, revealing that knockdown in neurons, intestines, hypodermis and muscles all reduced the lifespan extension of ADF (Figure S2a,b). Conversely, overexpression of *cpr‐2* in neurons, muscles, hypodermis and gut significantly extended the lifespan of N2 worms (Figure S2c,d). These findings indicate that *cpr‐2* and *cpr‐5* can function in multiple tissues to support the lifespan of worms.

### 2.4 Midlife‐stage ADF enhances the lysosome integrity through *cpr‐2* and *cpr‐5*

**FIGURE 3:**
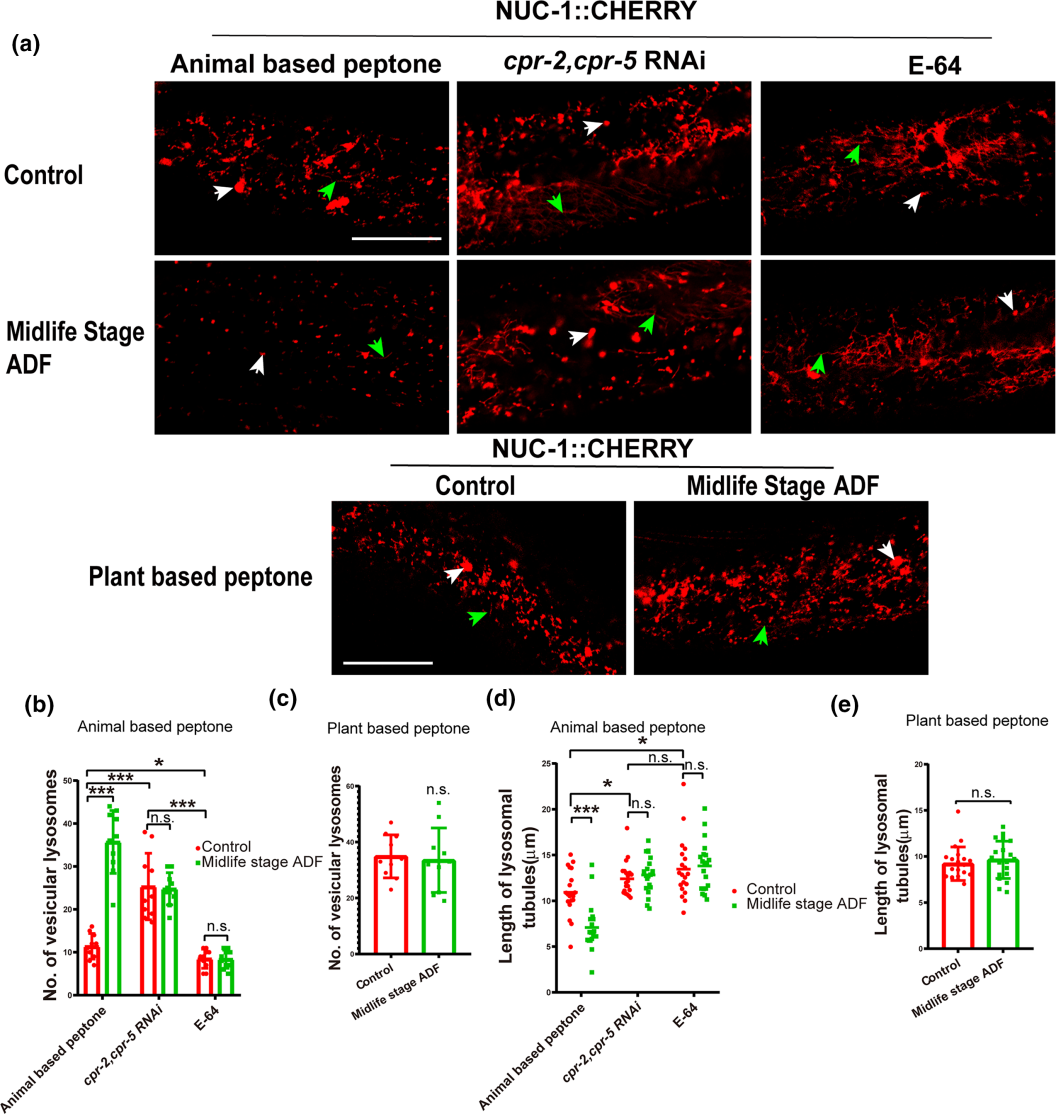
Alternative day fasting (ADF) performed on midlife age worms provided with animal‐based protein can increase the number of vesicular lysosomes and maintain tubular lysosomal structures in the hypodermis. (a) Confocal fluorescence images of lysosome morphology in the hypodermis of control worms, *cpr‐2/5* RNAi, and ADF worms expressing NUC‐1::CHERRY. White arrows indicate vesicular lysosomes; green arrows indicate lysosomal tubules. (b, c) Number of vesicular lysosomes, (d, e) tubule length were quantified. At least 10 animals were scored in each treatment for each repeat. Data are shown as mean ± SD. *p*‐Values were tested using *t*‐test. Scale bars: 25 μm. White arrow: Vesicular lysosome. Green arrow: Lysosome tube. E‐64: 20 μg/mL. *p < 0.05; ***p < 0.001. n.s., no significance.

Given that lysosome activity may be activated by ADF and *cpr‐2* is known to regulate lysosomal integrity (Zhang et al., 2023), we investigated lysosome activities in midlife age worms undergoing ADF. We examined lysosome morphology using the *qxIs257* (P*ced‐1*NUC‐1::CHERRY) reporter in midlife stage adults after ADF. Lysosomes appeared mainly as small puncta with short tubules in the hypodermis, with tubular lysosomal structures increased in both length and abundance, leading to the formation of an extensive tubular network during aging. We tested the properties of lysosomal morphology, including vesicular lysosomes. The number of vesicular lysosomes reduced gradually, but ADF slowed down this reduction (Figure 3a,b). Control worms exhibited longer tubular lysosomes (Figure 3a,d), whereas worms undergoing ADF displayed shorter tubular lysosomes (Figure 3a,d) by day 15. Additionally, knocking down *cpr‐2/5* and E‐64 treatments increased the decline of lysosome morphology and abolished the effects of ADF on the length of tubular lysosomes and the number of vesicular lysosomes (Figure 3a,b,d). Moreover, we investigated whether lysosome morphology was altered in midlife age worms cultured on soy peptone after ADF. Results showed that ADF failed to maintain lysosome morphology in these worms (Figure 3a,c,e), consistent with the conclusions of lifespan tests. We also compared the number of vesicular lysosomes in body wall muscle cells (Figure S3a,e) and intestinal cells (Figure S3c,g), our results showed that the worms treated with midlife stage ADF have more vesicular lysosomes than control worms, and knocking down *cpr‐2/5* and E‐64 treatments significantly reduced the vesicular lysosomes in these tissue cells, and abolished the effects of ADF. The lysosome morphology in the body wall muscle cells and intestinal cells of worms cultured on soy peptone was not changed by midlife ADF (Figure S3b,d,f,h).

**FIGURE 4:**
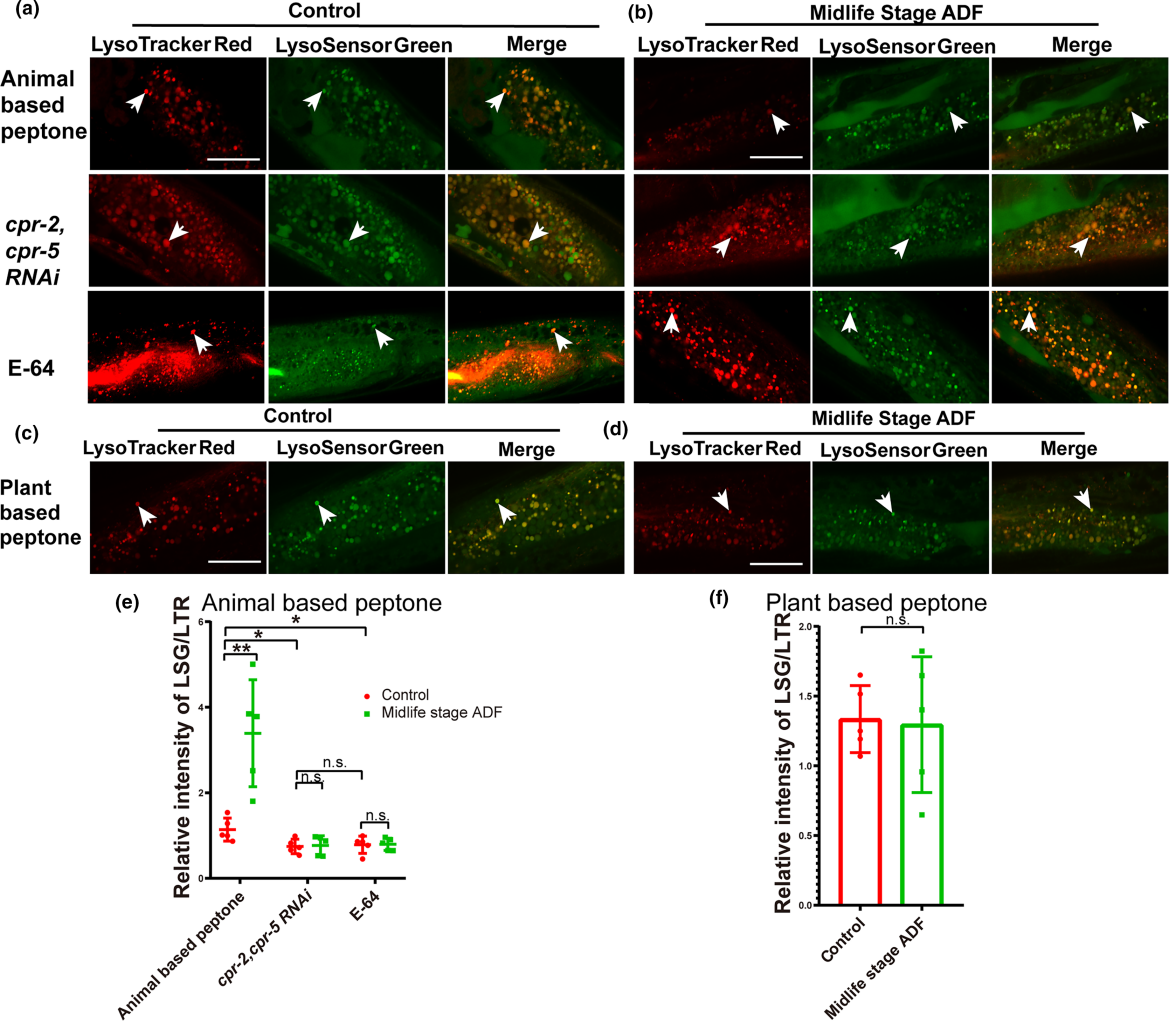
Alternative day fasting (ADF) performed on midlife age worms promotes intestinal lysosomal acidity. (a–d) Confocal fluorescence images of the intestine stained by LSG DND‐189 and LTR DND‐99. (e–f) The relative intensity of LSG/LTR in control and midlife stage ADF worms was quantified. At least five animals were scored in each treatment for each repeat. Data are shown as mean ± SD. *p*‐Values were calculated using *t*‐test. *p < 0.05, **p < 0.01. n.s., no significance. Scale bars: 25 μm. White arrow: Stained lysosome. E‐64: 20 μg/mL. LTR, LysoTracker Red; LSG, LysoSensor Green DND‐189.

The acidification, a crucial lysosomal property, undergoes alterations in worms as they age. To investigate whether ADF affects acidification in midlife worms, we conducted tests using LysoTracker Red (LTR) and LysoSensor Green DND‐189 (LSG, pKa 5.2), as detailed in previous studies (Baxi et al., 2017). LTR serves as a control to normalize dye intake, being less sensitive to increased acidity compared to LSG (Duvvuri et al., 2004). The fluorescence intensity ratio of LSG to LTR (LSG/LTR) quantifies lysosomal acidity. Our findings revealed an enhanced LSG/LTR ratio in the intestines of ADF‐treated worms compared to controls (Figure 4a,b,e), suggesting that ADF delays the decline in lysosomal acidity associated with aging. Moreover, knocking down *cpr‐2/5* and E‐64 treatments exacerbated the decline in lysosomal acidity and nullified the effects of ADF in maintaining lysosomal acidity (Figure 4a,b,e). We also assessed whether lysosomal morphology changed in midlife worms cultured on soy peptone after ADF. Results indicated no alteration in lysosomal acidity in these worms following ADF (Figure 4c,d,f).

**FIGURE 5:**
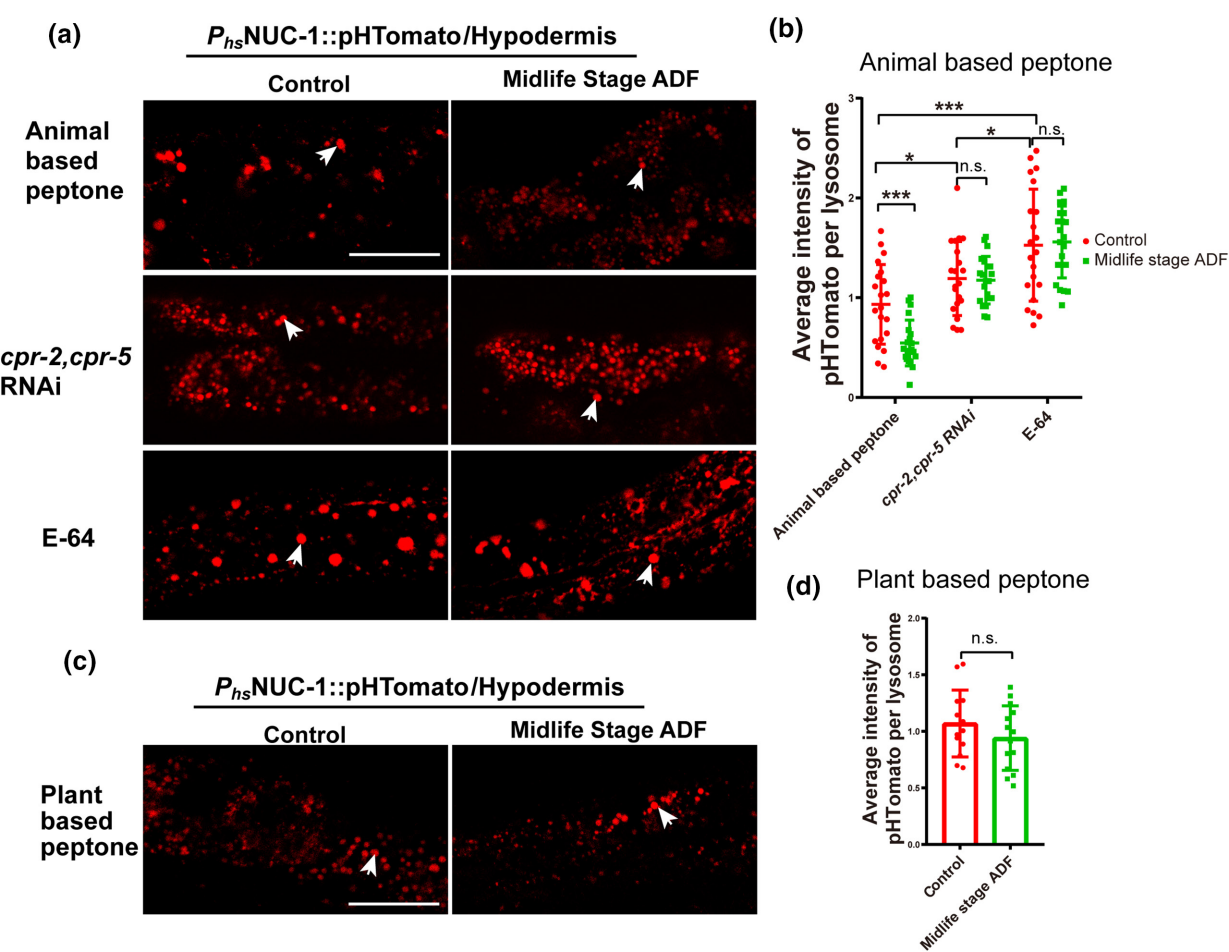
Alternative day fasting (ADF) performed on midlife age worms promotes hypodermal lysosomal acidity. (a, c) Confocal fluorescence images of the hypodermis in control and midlife stage ADF worms, expressing NUC‐1::PHTomato controlled by the heat‐shock (hs) promoter. The mean intensity of pHTomato per lysosome is shown in (b, d). At least 20 animals were scored in each treatment for each repeat. Data are shown as mean ± SD. *p*‐Values were calculated using *t*‐test. *p < 0.05, ***p < 0.001. n.s., no significance. Scale bars: 25 μm. White arrow: Lysosome. E‐64: 20 μg/mL.

To delve deeper into this phenomenon, we employed the heat‐shock promoter‐derived NUC‐1::pHTomato (a pH‐sensitive fluorescent protein) to assess acidity. With a pKa near 7.8, pHTomato exhibits increased fluorescence with rising pH (Li & Tsien, 2012). Twenty‐four hours of post‐heat shock treatment, we quantified the average fluorescence intensity of NUC‐1::pHTomato in each lysosome within the hypodermis. Significantly lower NUC‐1::pHTomato intensity was observed in ADF‐treated midlife worms compared to controls (Figure 5a,b). Conversely, knocking down *cpr‐2/5* and E‐64 treatments increased NUC‐1::pHTomato intensity and abolished the effect of ADF on NUC‐1::pHTomato intensity (Figure 5a,b). Additionally, we examined whether lysosomal acidity changed in midlife worms cultured on soy peptone after ADF. Results indicated that ADF failed to change lysosomal acidity under these conditions (Figure 5c,d), consistent with the conclusions drawn from lifespan tests.

In summary, these results suggest that ADF can maintain lysosomal activities and integrity, including acidity and morphology, by regulating the expression of *cpr‐2* and *cpr‐5*. Furthermore, they indicate that ADF does not influence lysosomes in worms cultured on soy peptone.

### 2.5 ADF‐induced lifespan extension through lysosome activity is regulated by *eat‐2*, *skn‐1*, and *daf‐16*

**FIGURE 6:**
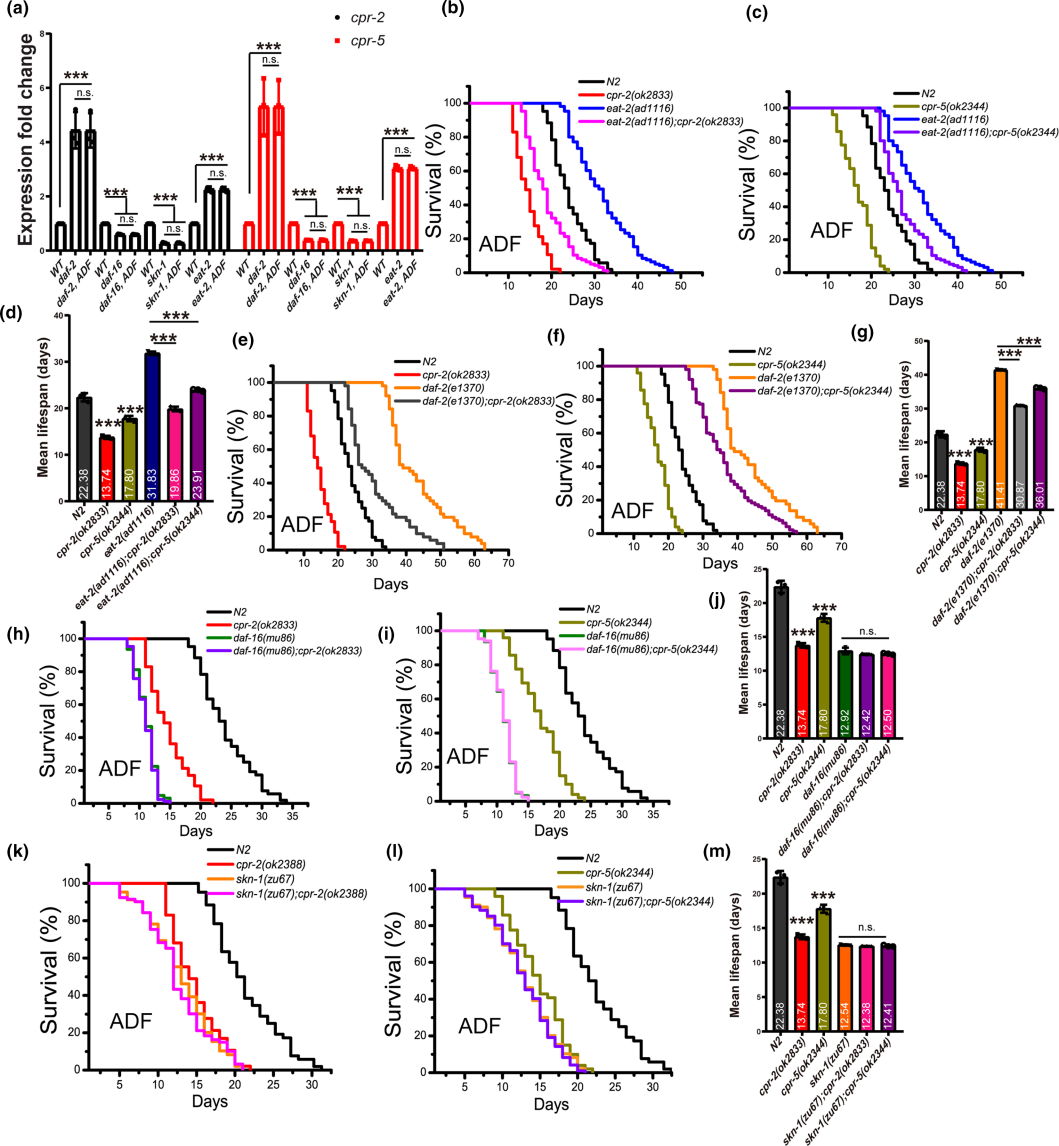
Alternative day fasting (ADF) regulates the expression of *cpr‐2/5*, which is related to multiple longevity signaling pathways. (a) The expression of *cpr‐2* and *cpr‐5* was affected by ADF in multiple mutants, as tested by real‐time PCR. ***p < 0.001. *p*‐Values were calculated using a *t*‐test. WT: N2. (b, c) Mutation of *cpr‐2* and *cpr‐5* reduced the lifespan extension of *eat‐2* mutants when subjected to ADF in midlife age. (d) Summary of the lifespan data. (e, f) Mutation of *cpr‐2* and *cpr‐5* also reduced the lifespan extension of *daf‐2* mutants when subjected to ADF in midlife age. (g) Summary of the lifespan data. (h, i) Mutation of *cpr‐2* and *cpr‐5* failed to further reduce the short lifespan of *daf‐16* mutants when subjected to ADF in midlife age. (j) Summary of the lifespan data. (k, l) Mutation of *cpr‐2* and *cpr‐5* also failed to further reduce the short lifespan of *skn‐1* mutants when subjected to ADF in midlife age. (m) Summary of the lifespan data. Each survival curve is representative of at least three independent repeats. P values were determined using the log‐rank test. ***p < 0.001. n.s., no significant difference. The detailed data of lifespan was summarized in Table S3 in supporting information.

The cathepsin protease‐encoding genes are crucial for lysosomal acidity and integrity (Sun et al., 2020; Yang & Wang, 2021; Zhang et al., 2023). The up‐regulation of these genes depends on DAF‐16/FOXO in the IIS and SKN‐1/NRF2 transcription factors (Sun et al., 2020). Additionally, the *eat‐2*‐related caloric restriction pathway controls lysosome activity. We observed that the expression of *cpr‐2* and *cpr‐5* was affected by *daf‐2, daf‐16*, *skn‐1*, and *eat‐2* (Figure 6a). Subsequently, we investigated whether these pathways affect the lifespan extension induced by ADF. We found that ADF treatment in midlife age *eat‐2* mutants had a limited effect on lifespan extension (Figure S4a,e), while *daf‐2* mutants exhibited similar limited effects (Figure S4b,e). Moreover, *daf‐16* and *skn‐1* mutants almost completely abolished the lifespan extension induced by ADF (Figure S4c–e). Furthermore, we observed that the translocation of DAF‐16 into the nucleus was significantly enhanced by ADF treatment (Figure S4f). Using the *skn‐1* reporter *gst‐4*::*gfp*, we confirmed that the intensity of *gst‐4*::*gfp* was higher in ADF‐treated worms compared to controls (Figure S4g), suggesting that ADF activation of lysosomes also requires *skn‐1*. These findings suggest that ADF‐induced lysosome activity to extend lifespan requires the IIS pathway. Taken together, these results confirm that ADF‐induced lysosome activity to improve the lifespan of midlife‐aged worms depends on DAF‐16, SKN‐1, and EAT‐2.

To further confirm this conclusion, we investigated whether the expression of *cpr‐2* and *cpr‐5* induced by ADF in midlife‐aged worms is affected by *daf‐16*, *skn‐1*, and *eat‐2*. Our results showed that ADF failed to further change the expression of *cpr‐2* and *cpr‐5* in *daf‐2*, *daf‐16*, *skn‐1*, and *eat‐2* mutants (Figure 6a). In order to further confirm whether *cpr‐2* and *cpr‐5*‐regulated lifespan extension requires these pathways when worms are treated with midlife stage ADF, we conducted epistasis analysis. We found that the *cpr‐2* and *cpr‐5* mutation partially decreased the lifespan of *eat‐2* (Figure 6b–d) and *daf‐2* mutants (Figure 6e–g), but failed to further reduce the shortened lifespan of *daf‐16* (Figure 6h–j) and *skn‐1* mutants (Figure 6k–m). These results further confirm that the function of *cpr‐2* and *cpr‐5* in lifespan extension depends on *eat‐2*, *skn‐1*, and *daf‐16*, and also suggest that *daf‐16* and *skn‐1* may be more important in regulating the function of *cpr‐2* and *cpr‐5*. Our findings suggest that the efficacy of ADF is influenced by insulin/insulin‐like growth factor signaling (IIS), EAT‐2, and SKN‐1 pathways, which support lifespan.

### 2.6 ADF induces lysosomal lipophagy regulated by HLH‐30

Lysosomes serve as central regulators of cellular metabolism and the fasting response, functioning as incinerators to facilitate autophagy. The master growth regulator mTORC1 orchestrates the protein cascade linking nutrient sensing and acute regulation of autophagy and lysosomal function. A crucial target of mTORC1 kinase activity at the LYNUS complex is the transcription factor TFEB, which governs lysosomal biogenesis and autophagy (Settembre et al., 2012). mTOR activation has been shown to modulate TFEB activity (Settembre et al., 2012), crucial for activating lysosomal gene expression, autophagosome biogenesis, and autophagosome‐lysosome fusion, ultimately leading to the degradation of long‐lived proteins within lysosomes. Fasting‐induced TFEB activation results in the transcriptional stimulation of lysosomal machinery. In *C. elegans*, the TFEB homologue is HLH‐30. We investigated whether the effects of ADF on lifespan extension operate through HLH‐30. Our results revealed that *hlh‐30* mutation significantly decreased the lifespan extension induced by ADF in midlife‐aged worms (Figure S5a,b). Additionally, ADF significantly enriched the presence of HLH‐30 in intestinal nuclei (Figure S5c). Since ADF alters the feeding‐fasting pattern in worms, we examined the role of mTOR in ADF's function. Knocking down *let‐363* (mTOR complex encoding gene) partially decreased the lifespan extension in ADF‐treated midlife‐aged worms (Figure S5d,i). However, our RNA‐seq data did not show significant changes in autophagy‐related genes in ADF worms compared to controls. To further investigate whether autophagy is affected by ADF in midlife‐aged worms, we employed autophagy reporters LGG‐1::GFP and SQST‐1::GFP. Our results indicated that autophagy is not affected by ADF (Figure S5e,f).

HLH‐30 promotes lifespan by enhancing lysosomal lipophagy mainly through *lipl‐1* and *lipl‐3* (O'Rourke & Ruvkun, 2013). Our findings showed a significant upregulation of *lipl‐3* expression in midlife‐aged worms treated with ADF (Figure S5g). Subsequently, knocking down *lipl‐3* significantly decreased the lifespan extension induced by ADF in midlife‐aged worms (Figure S5h,i). Furthermore, we observed a reduction in fat storage in ADF‐treated midlife‐aged worms compared to controls (Figure S5j,k), suggesting that ADF may induce lipophagy to reduce the fat accumulation. These results suggested that Midlife ADF induces the expression of lysosomal protease genes *cpr‐2/5*, thereby preserving the integrity of lysosomes. These beneficial effects of ADF appear to be closely linked to the integrity and digestive functions of lysosomes. However, it's noteworthy that even though autophagy is not affected by midlife age ADF, it induces *hlh‐30*‐regulated lipid metabolism in lysosomes through lipophagy.

### 2.7 Midlife‐stage ADF reduces aggregated proteins during aging

**FIGURE 7:**
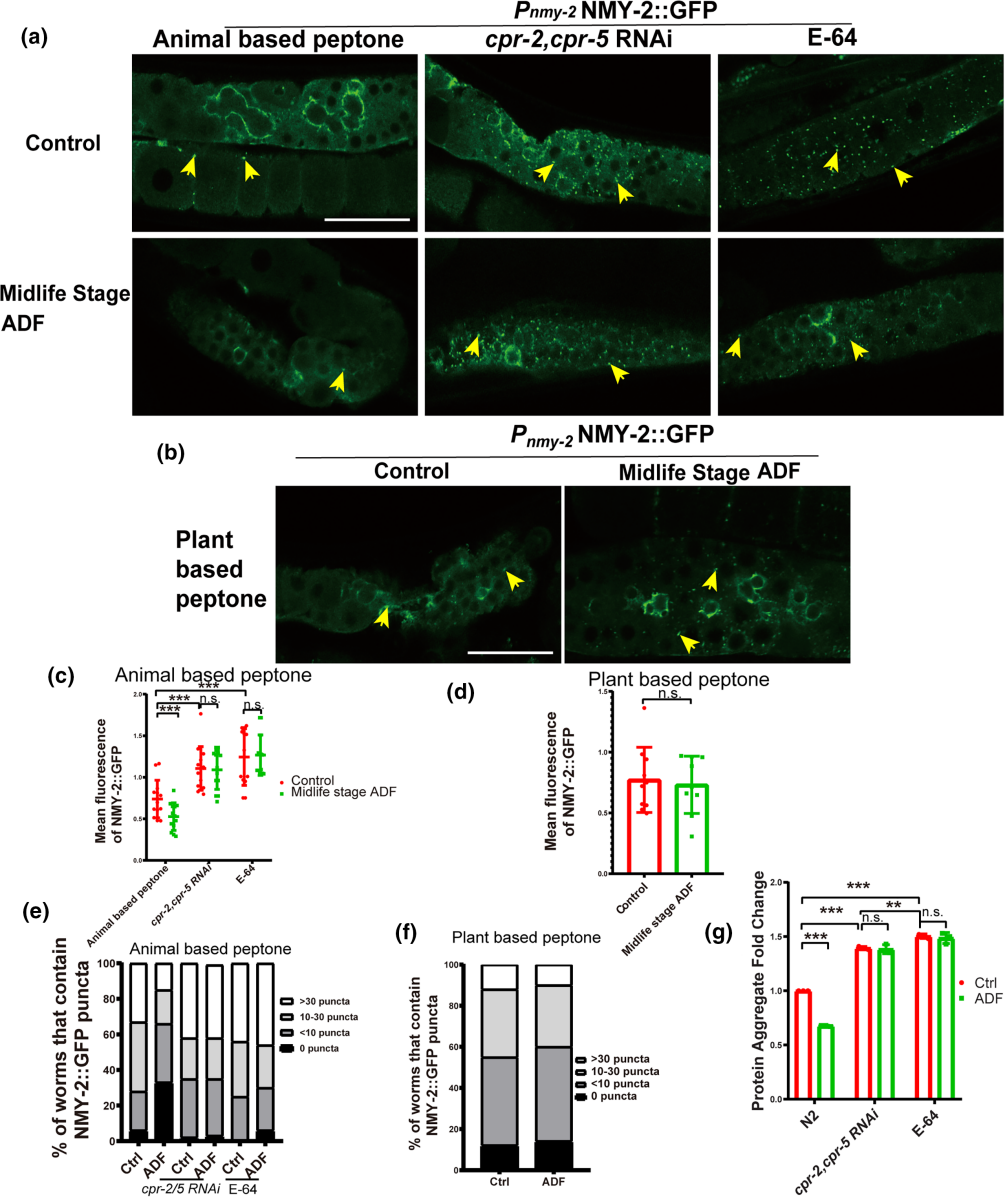
Lysosome activity is crucial for the clearance of aggregate‐prone proteins and lifespan extension. (a, b) Confocal fluorescence images of oocytes in control, *cpr‐2/5* RNAi, and alternative day fasting (ADF) worms expressing NMY‐2::GFP. Yellow arrows indicate NMY‐2::GFP puncta. Scale bars: 25 μm. (c, d) The mean intensity of NMY‐2::GFP. At least 50 animals were scored in each treatment. *p*‐Value was calculated using a *t*‐test. ***p < 0.001. n.s., no significance. (e, f) The number of NMY‐2::GFP puncta was quantified. At least 10 animals were scored in each treatment for each repeat. Ctrl: Control worms. ADF: Worms treated with midlife stage ADF. (g) Total protein aggregation was tested using an in vitro method. The experiment was repeated at least three times. Ctrl: Control worms. ADF: Worms treated with midlife stage ADF. Data show Mean ± SD, *t*‐test. **p < 0.01, ***p < 0.001. n.s.: no significance. E‐64: 20 μg/mL.

Aged worms accumulate more aggregated proteins, reducing the lifespan of animals. Lysosomes serve as degradation centers for these molecules. To investigate whether ADF affects lysosome degradation activity, we employed in vitro and in vivo methods. Additionally, we utilized a lysosome degradation activity reporter, *Pnmy‐2*NMY‐2::GFP, to assess in vivo lysosome activity. Our results indicated a significant reduction in the mean intensity and numbers of NMY‐2::GFP puncta in old worms treated with ADF during midlife‐aged worms (Figure 7a,c,e), suggesting enhanced lysosome degradation activity during ageing. Moreover, knocking down *cpr‐2/5* and E‐64 treatments significantly increased the mean intensity and numbers of NMY‐2::GFP puncta and abolished the effect of ADF (Figure 7a,c,e). However, degradation activity in soy peptone‐cultured worms was not affected by ADF (Figure 7 b,d,f). Furthermore, we observed a decrease in aggregated proteins in old worms treated with ADF during midlife‐aged worms, knocking down *cpr‐2/5* and E‐64 treatments significantly increased the aggregated proteins and abolished the effect of ADF (Figure 7g). Lysosome function is also reported to be crucial in neurodegenerative diseases (Udayar et al., 2022). We investigated whether ADF‐induced lysosome degradation activity could degrade aggregated toxic proteins such as *α‐synuclein* (*α‐syn*) or amyloid‐beta (Aβ). Our results demonstrated a reduction in *α‐syn* levels and an increase in the thrashing rate of worms in worms treated with ADF during midlife‐aged worms (Figure S6), along with reduced neuron damage in head neurons (Figure S7). Additionally, in an Alzheimer's disease model, ADF reduced Aβ accumulation and decreased paralysis percentages in worms (Figure S8). However, the effects of ADF on protein degradation in neuronal disease models were observed only in worms cultured on animal‐based protein sources, not plate‐based soy peptone (Figure S6–S8). In conclusion, midlife‐stage ADF enhances lysosome integrity and activity, reducing the accumulation of toxic aggregated proteins in worms.

## 3 DISCUSSION

Using *C. elegans* as a model, we subjected them to extreme ADF to investigate its effects on lifespan. Our study advances the understanding of ADF effects. Previously, it was generally believed that low‐calorie intake was critical for affecting lifespan and health, and that the types or quantities of foods consumed on feast days would not affect the function of ADF. However, we found that animals cultured on media containing plant‐based protein sources were not affected by ADF, and that the lifespan extension induced by ADF may only apply to those consuming animal‐based protein‐rich diets. Protein types and quality are known to affect animal health (Solon‐Biet et al., 2015), and proteins are digested into different peptides, while the varied amino acid chains in different protein sources may influence ADF function. Recent studies have linked plant‐based dietary patterns to the mobility of older people (Chen et al., 2022). Worms cultured on soy peptone live longer than those cultured on animal‐based peptone, yet we found that plant‐based protein sources failed to induce the effects of ADF on lifespan and aging. Plants contain abundant and varied cysteine proteases (David Troncoso et al., 2022). In our study, ADF induced the activity of cysteine‐type endopeptidases encoded by *cpr‐2* and *cpr‐5* in worms cultured on animal‐based protein source media. We hypothesize that worms cultured on soy peptone have already adapted to these protein diets containing cysteine proteases from plants, and ADF may not further evoke the expression of *cpr‐2* and *cpr‐5*. Our results confirmed that *cpr‐2/5* was not affected by ADF in young and midlife worms cultured on plant‐based peptone (Figure S9a,b). Additionally, plant‐based proteins contain high carbohydrate and sugar content, which may also affect the function of ADF. In this study, we only found that protein as a primary food resource can affect the function of ADF on lifespan; other macromolecules such as fatty acids and sugars were not considered, warranting further investigation in the future.

The ages of individuals in ADF human trials are also random, making it uncertain which age stage is more functional for performing ADF. Our study clarified that ADF performed on young adults can be harmful to normal development, especially to the reproductive system and embryonic development of progeny. This is probably due to *cyd‐1* and *cfz‐2* expression being affected by ADF specifically in young stage worms cultured on plant‐ or animal‐based peptone (Figure S9a–c). ADF performed on older animals does not have any positive effects on lifespan extension, although whether other health aspects are affected by ADF may need further investigation. We found that ADF performed on midlife age worms is sufficient to extend lifespan, which may be mainly related to the maintenance of lysosome function during aging. We did find that the lysosome‐encoding gene *cpr‐5* was also upregulated in young stage worms (Figure S9d); however, ADF caused serious developmental issues, leading to the premature death of many individual worms and an unhealthy lifespan. Therefore, the maintenance of lysosome function during aging is not sufficient to support their survival, or the *cpr‐5*‐regulated lysosome function is not sufficient to support the lives of these worms. As we found that *cpr‐2* is stronger than *cpr‐5*, suggesting that *cpr‐2*‐regulated lysosome integrity may play a pivotal role in aging controlled by ADF.

Recent studies have reported the importance of *cpr‐2* activity in preserving lysosomal membrane integrity (Zhang et al., 2023), consistent with our findings. Additionally, we observed that *cpr‐5* was also up‐regulated by ADF to support lifespan extension, although our results suggest that *cpr‐2* plays a more dominant role in regulating lysosomal function. Our study indicates that both *cpr‐2* and *cpr‐5* play roles in lysosome activity, suggesting they may have non‐redundant effects. Considering each cathepsin gene may have unique biological functions (Honey & Rudensky, 2003; Peng et al., 2017; Xie et al., 2023), *cpr‐5* and *cpr‐2* may regulate lysosome activity diversely. The activity of *cpr‐2* cathepsin in lysosomes is regulated by *clh‐6* (Zhang et al., 2023), which may support this speculation.

During periods of feeding and fasting, cellular homeostasis may be restored, depending on intact autophagy and lysosomal function (Godar et al., 2015). Lysosome function during fasting is closely linked to autophagy (Mani et al., 2018). Our study suggests that autophagy is not affected by ADF‐induced lysosome activity mediated by *cpr‐2* and *cpr‐5*. Current work has reported that CLH‐6 regulates *cpr‐2* to facilitate substrate digestion, protecting lysosome membrane integrity through endocytosis (Zhang et al., 2023), which is consistent with our results. This may suggest that ADF‐induced lifespan extension through *cpr‐2*‐regulated lysosome endocytosis rather than autophagy to degrade aggregated proteins. However, the effects of ADF on HLH‐30‐regulated lipolysis in midlife worms aligns with previous findings indicating that HLH‐30 promotes lifespan by enhancing lysosomal lipolysis, acritical process for lipid homeostasis (O'Rourke & Ruvkun, 2013). The lysosomal acid lipase facilitates the breakdown of triglycerides and cholesteryl esters within lysosomes, which is essential for regulating lipid metabolism in the organism. Moreover, lysosomes play a crucial role in lifespan and age‐related diseases by degrading macromolecules through processes various processed such as endocytosis, phagocytosis, and autophagy (Sun et al., 2020; Yang & Wang, 2021).

The central protein cascade that links nutrient sensing with the regulation of autophagy and lysosomal function is orchestrated by mTORC1 (Yang & Wang, 2021). Activation of mTOR inhibits TFEB activity, which in turn activates genes responsible for autophagosome biogenesis and fusion with lysosomes, thereby linking autophagy with lysosomal function (Yang & Wang, 2021). In *C. elegans*, HLH‐30, a homologue of TFEB, contributes to longevity, particularly in scenarios where autophagy‐lysosome pathways are compromised due to various factors such as altered food intake, mitochondrial respiration, insulin/IGF‐1 signaling (IIS), TOR signaling (Lapierre et al., 2013). However, the cyclic activation and deactivation of lysosome‐mTOR during ADF or CR may not solely depend on intact autophagy (Honjoh et al., 2009). Our findings suggest that the mTOR pathway is indeed related to the effects of ADF on lifespan extension, possibly acting through the IIS pathway, consistent with prior reports indicating that TOR signaling is necessary for the fasting‐induced downregulation of insulin‐like peptides (Honjoh et al., 2009). Additionally, lysosomal hydrolases, particularly cysteine proteases, play a crucial role in molecule digestion, each likely serving distinct functions (Turk, 2001).

Lysosomal activity, crucial for maintaining protein homeostasis by digesting aggregated proteins, tends to decline with age, contributing to age‐related diseases (Carmona‐Gutierrez et al., 2016). In worms, fasting triggers proteostasis pathways (Longo & Anderson, 2022), revealing a protective aspect to metabolic changes during fasting and underscoring the importance of recycling damaged proteins. Our research demonstrates that ADF activates and sustains lysosomal degradation activity, significantly reducing the total aggregated proteins in older worms after ADF treatment. This suggests that ADF during midlife may benefit worms in old age by preserving protein homeostasis as they age. Given that *cpr‐2* and *cpr‐5* are widely expressed in neurons (Durham et al., 2021; Lockhead et al., 2016), and genes involved in controlling lysosomal function have been linked to prevalent idiopathic neurodegenerative diseases, including Alzheimer's disease, Parkinson's disease, and amyotrophic lateral sclerosis (Udayar et al., 2022), our study also investigated the effect of ADF on neurodegenerative disease models in nematodes. We observed that ADF in midlife age worms effectively degraded aggregated proteins in these models, improving health and reducing the burden of neurological disease inducers. ADF administered to midlife‐aged worms cultured on animal‐based protein sources exhibits enduring effects throughout aging. It effectively delays the age‐related decline in lysosomal integrity observed in older worms. This preservation of lysosomal function enhances protein homeostasis and reduces protein aggregation during aging and can mitigate disease markers and improve the health of neurodegenerative model worms, which potentially offering valuable insights into combating age‐related diseases, such as neurodegenerative diseases. This work suggests that ADF in midlife age animals may confer benefits or delay the onset of neuronal diseases.

## 4 MATERIALS AND METHODS

### 4.1 *C. elegans* strains

Worms were grown and maintained on standard nematode growth medium (NGM) at 20 °C as previously described (Brenner, 1974), unless otherwise indicated. The NGM plates were preseeded with OP50 *Escherichia coli*. The *C. elegans* strains used in this study were acquired from the Caenorhabditis Genetics Center (CGC), which is supported by the NIH Office of Research Infrastructure Programs (P40OD010440).

Strains used in this study: wild‐type: N2, JK2505:*cyd‐1*(*q626*); *him‐5*(*e1490*), RB1162:*cfz‐2*(*ok1201*), RB2129:*cpr‐2*(*ok2833*), RB1810:*cpr‐5*(*ok2344*), CB3844: *fem‐3*(*e2006*), XW13734:*qxIs612*(*PhsNUC‐1*::*sfGFP*::*CHERRY*), XW10197:*qxIs468*(*Pmyo‐3LAAT‐1*::*GFP*), XW11282:*qxIs520*(*Pvha‐6LAAT‐1*::*GFP*), XW19180:*qxIs750*(*PhsNUC‐1*::*pHTomato*), CB1370:*daf‐2*(*e1370*), DA1116:*eat‐2*(*ad1116*), EU1:*skn‐1*(*zu67*)*/nT1* [*unc‐α*(*n754*)*let‐α*], CF1038:*daf‐16*(*mu86*), CL2166:*dvIs19* [(*pAF15*)*gst‐4p*::*GFP*::*NLS*], JIN1375:*hlh‐30*(*tm1978*), JJ1473:*zuIs45*(*Pnmy‐2NMY‐2*::*GFP*), BZ555:*egIs1* [*dat‐1p*::*GFP*], NL5901:*pkIs2386* [*unc‐54p*::*α‐syn*::*YFP + unc‐119*(+)], GMC101:[(*Punc‐54*::*A‐beta*::*unc‐54 3Prime UTR; Pmtl2*::*GFP*)], OH16024:*daf‐16*(*ot971[daf‐16*::*GFP*]), MAH240:*sqIs17* [*hlh‐30p*::*hlh‐30*::*GFP + rol‐6*(*su1006*)], DA2123: *adIs2122* [*lgg‐1p*::*GFP*::*lgg‐1 + rol‐6*(*su1006*)], HZ946: *bpIs151* [*sqst‐1p*::*sqst‐1*::*GFP + unc‐76*(+)], TU3401: *sid‐1*(*pk3321*); *uIs69 V*, VP303: *rde‐1*(*ne219*) *V*; *kbIs7*, WM118: *rde‐1*(*ne300*) *V*; *neIs9 X*, NR222: *rde‐1*(*ne219*) *V*; *kzIs9*.

### 4.2 Survival and lifespan assays

The standard method to prepare NGM plates, eggs and synchronized worms was used (Zheng & Chin‐Sang, 2016). To prepare the NGM plates: In a 2 L Erlenmeyer flask, mix 3 g NaCl, 17 g agar, and 2.5 g peptone (plant‐based peptone: peptone from soybean, CAS No.:91079‐46‐8, Sigma; animal‐based peptone: peptone from meat, CAS No.:91079‐38‐8, Sigma). Add 975 mL H_2_O. Cover the mouth of the flask with aluminum foil and autoclave for 50 min. Cool the flask in a 55°C water bath for 15 min. Add the following to the cooled solution:1 mL 1 M CaCl_2_, 1 mL 5 mg/mL cholesterol in ethanol, 1 mL 1 M MgSO_4_ and 25 mL 1 M KPO_4_ buffer. Swirl to mix well. Using sterile procedures, dispense the NGM solution into petri plates using a peristaltic pump, filling the plates two‐thirds full of agar. Leave the plates at room temperature for 2–3 days before use to allow for the detection of contaminants and the evaporation of excess moisture.

To prepare eggs and synchronized worms, washing gravid adults from 1 to 3 NGM plates (6 cm each) with 1 mL M9 buffer and transfer to a 1.5 mL centrifuge tube. Centrifuge at 1000×*g* for 1 min to pellet the worms. Aspirate the M9 buffer and resuspend the worm pellet in 1 mL of bleach solution (Mix 0.5 mL 5 N NaOH with 1 mL 5% solution of sodium hypochlorite). Agitate for 1 min. Centrifuge at 1000×*g* for 1 min, aspirate the solution, and resuspend the pellet in another 1 mL of bleach solution. Agitate/mix until the worm bodies are no longer visible, leaving only the eggs (<3 min). Centrifuge the embryos at 3000×*g* for 1 min. Aspirate off the bleach solution, being careful not to disturb the egg pellet. Perform a minimum of three washes with 1 mL M9 buffer each time, spinning at 3000×*g* for 1 min and aspirating to the pellet. After the final wash, resuspend the eggs in 1 mL M9 buffer to achieve a concentration of <10 eggs/μL. Place the tube on a rocker/shaker at 20°C for 15 h to let the eggs hatch. Then transfer the synchronized L1 worms into the maintenance NGM plates and cultured at 20°C.

The lifespan of adult worms was analyzed by randomly selecting worms at the L4 stage from maintenance culture plates and transferring them to new culture plates sprinkled with OP50 *E. coli* to continue cultivation at 20°C. To eliminate interference caused by the next generation of larvae, we regularly transferred the worms to new NGM plates sprinkled with food, ensuring consistency in comparisons. The day when the worms grew into adults was considered the starting point of the experiment, and the survival status of the worms was checked daily thereafter. Worms were deemed dead if they no longer responded to a light touch. Each strain underwent at least three rounds of independent experiments, with each round including more than 60 worms. We used the Kaplan–Meier method to calculate the average survival rate and assessed the significance of differences in survival rates using the log‐rank test.

### 4.3 Alternate‐day fasting

Alternate‐day fasting was implemented for worms at different developmental stages. The specific procedure involved transferring the worms scheduled for fasting to an empty NGM plate. M9 buffer was then dropped onto the plate to allow the worms to swim freely in the liquid, aiming to remove as much OP50 bacteria from their bodies as possible. This process was repeated three times. Afterwards, the worms were transferred to a new empty NGM plate and cultured for 1 day. The next day, the worms were moved to a plate sprinkled with OP50. The entire cycle lasted for 7 days, with feeding on the first day and fasting on the second day, alternating in this manner.

### 4.4 Ovulation time and hatching time

To investigate the egg‐laying and hatching conditions of *C. elegans* in the young stage after ADF, we transferred single worms synchronized to the L4 stage onto sterile NGM plates and cultured them at 20°C. Each worm was cultured in a petri dish for 24 h, and the number of eggs laid was recorded every half an hour. We began calculating the hatching rate when the eggs in the control group started to hatch. Each group consisted of 30 worms.

### 4.5 Microscopy and imaging analysis

A 63x objective (PlanNeofluar NA1.30) was used with Immersol 518F oil (Carl Zeiss). Confocal images were captured by a Zeiss 880 inverted laser scanning confocal microscope with 488 nm (emission filter BP 503–530) and 543 nm (emission filter BP 560–615) lasers. Images were processed and viewed using Zen software (Carl Zeiss). Observations were made using a Nikon laser confocal high‐resolution live‐cell imaging system with a 40× objective lens. All images were taken at 20°C.

### 4.6 GFP fluorescence

To carry out fluorescence imaging, nematodes from different treatment groups were first washed with M9 buffer, then anesthetized with M9 salt solution containing 25 mM levamisole hydrochloride, and placed on a 2% agar pad. Subsequently, the worms were observed using the Leica M165 FC fluorescent stereo microscope.

### 4.7 Quantification of lysosomal tubule length

The length of tubular lysosomes was measured as previously described (Sun et al., 2020). Fluorescence images of adult *C. elegans* from different treatment groups, labeled with NUC‐1::CHERRY, were captured using a laser scanning confocal microscope (Carl Zeiss). The length of tubular bodies expressing NUC‐1::CHERRY in each worm was quantified using ImageJ software. If tubular lysosomes intersected, they were counted as two separate tubules.

### 4.8 Quantification of lysosome number

The method for detecting the number of lysosomes was consistent with that previously described (Sun et al., 2020). The number of vesicular lysosomes expressing NUC‐1::CHERRY in each field was quantified using ImageJ software. In each treatment group, at least eight worms were analyzed.

### 4.9 LysoSensor green and LysoTracker staining

The acidity of lysosomes was measured using the same method as previously described (Duvvuri et al., 2004; Sun et al., 2020). Nematodes from different treatment groups were soaked in 80 μL of M9 buffer solution containing LSG and LysoTracker Red DND 99 (Invitrogen, Oregon, USA) at a concentration of 10 μM for intestinal staining. After staining for 1 h in the dark at 20°C, the nematodes were transferred to NGM plates containing fresh OP50 and allowed to recover for 1 h under the same dark conditions at 20°C before being assayed. The relative intensity of LSG/LTR was quantified using ImageJ software.

### 4.10 Quantification of NUC‐1::pHTomato intensity

The fluorescence intensity of the pH‐sensitive fluorescent protein was measured using the same method as previously described (Sun et al., 2020). After incubation at 33°C for 30 min followed by 24 h at 20°C, adult *C. elegans* expressing PhsNUC‐1::pHTomato were examined. The average intensity of pHTomato within each lysosome under the skin was measured using ImageJ software.

### 4.11 Quantification of NMY‐2::GFP Intensity and number of puncta

The aggregation content of NMY‐2 protein was measured using a previously reported method (Bohnert & Kenyon, 2017; Sun et al., 2020). Fluorescence images of *C. elegans* from different treatment groups expressing NMY‐2::GFP were captured using a laser scanning confocal microscope (LSM 980, Carl Zeiss). We measured the fluorescence intensity of the oocytes using ImageJ software and manually counted the number of NMY‐2::GFP puncta in the oocytes.

### 4.12 Number of LGG‐1::GFP or SQST‐1::GFP foci

A reported experimental method was employed (Zhang et al., 2015). Fluorescence images of *C. elegans* expressing LGG‐1::GFP and SQST‐1::GFP were captured using a laser scanning confocal microscope (LSM 980, Carl Zeiss AG). Subsequently, the number of LGG‐1::GFP and SQST‐1::GFP fluorescent spots within the cells was manually counted using ImageJ software.

### 4.13 Plasmid construction

The sequences of the *cpr‐2* and *cpr‐5* genes, as well as the promoters of *ges‐1, rgef‐1*, *dpy‐7*, and *myo‐3*, were amplified from N2 *C. elegans* genomic DNA using Phusion High‐Fidelity PCR Master Mix (Thermo Fisher) and the following primers:

**Table:** 

*cpr‐5* gene sequence:
Forward AATTGCTAGCATGTGGAAGCTCTCCGCTATTCTTCTCGTGGC
Reverse AATTGGTACCTTAGTTGTGACGAGCCAAGTCTGGAATT
*cpr‐5* promoter:
Forward AATTCCCGGGTTTTCAGCTTTGAATTGAGGTTTGTGTGCGGA‐
Reverse AATTGCTAGCTATGAGAGAAGTGTCTGCGAAGGTTCGCGTC
*cpr‐2* gene sequence:
Forward: AATTGCTAGCATGGTAAGTTTTTCATTTAACCAACGAG
Reverse: AATT GATATCTTATCTCGGCAATCCAGCGACAATACG
*cpr‐2* promoter:
Forward: AATTCCCGGGCATACAGTACTACTACAGTACCCCCACAGTA
Reverse: AATTGCTAGCCGTAAAAGCCAGACTGAGCTCTGCCTGTGTC
*ges‐1* promoter:
Forward: AATTCCCGGGAAACTCCGAACTATGATGACG
Reverse: AATTGCTAGCCTGAATTCAAAGATAAGATATGT
*rgef‐1* promoter:
Forward: AATTCCCGGGTTTCCGTCAATTCTACCTCCCCAATCTTCA
Reverse: AATTGCTAGCCGTCGTCGTCGTCGATGCCGTCTTCACG
*myo‐3* promoter:
Forward: AATTAAGCTTGGTCTGGCCGCAAAAAGGTTTATGG
Reverse: AATTCCCGGGTTCTAGATGGATCTAGTGGTCGTGGG
*dpy‐7* promoter:
Forward: AATTCCCGGGTCCCTACCAATTGAAAATTCAGAAACCCATGAA
Reverse: AATTGCTAGCTTATCTGGAACAAAATGTAAGAATATTCTTA

The amplified PCR products were digested and inserted into the worm expression plasmid L2518 by using a standard method (Zheng et al., 2018). A plasmid with the injection marker (roller) pJRK 248 (Addgene) was injected into worms using standard microinjection methods (Mello et al., 1991). Each injected strain had at least three stable lines.

### 4.14 RNA sequencing

Samples of nematodes from the different treatment groups were collected, and the quality of total RNA was assessed using a NanoDrop ND‐1000 (Agilent Technologies). Subsequently, library construction was performed with the KAPA Stranded RNA‐Seq Library Prep Kit (Illumina), and the quality of the libraries was verified using the Agilent 2100 Bioanalyzer (Agilent Technologies). Transcriptome alignment and quantification were carried out with the HISAT2 software, referencing the *C. elegans* genome version WBcel235. Using the Ballgown package from the R project, we calculated the fragments per kilobase of transcript per million mapped fragments (FPKM) to identify differentially expressed genes. Additionally, we conducted KEGG pathway and GO function enrichment analyses using the latest version of R or Python developed by Aksomics Inc. Finally, heatmaps were generated based on the logarithmic average of the FPKM values for each group (i.e., log_2_ (FPKM +1)).

### 4.15 RNAi treatment

RNAi experiments were conducted using standard culturing methods (Rual et al., 2004) and the Horizon RNAi library. In most experiments, 3–5 L4 stage larvae (P0) were cultured on RNAi plates, and their F1 progeny were assessed from the late larval to the pupal stages. The worms were fed HT115 bacterial strains containing either an empty control vector (L4440) or an RNAi construct expressing double‐stranded RNA of the target gene (vector L4440). Phenotypes were examined in the adult stage of the same generation.

### 4.16 Quantitative real‐time PCR (qRT‐PCR)

Total RNA was extracted from collected nematode samples using RNAiso Plus (Takara) and then converted to cDNA using the high‐capacity cDNA reverse transcription kit (Applied Biosystems). The qRT‐PCR experiments were performed using Power SYBR Green PCR Master Mix (Applied Biosystems) and the ABI 7500 system. The relative expression levels of genes were determined using the 2^−ΔΔCT^ method and normalized to the expression of the *cdc‐42* gene. *p*‐Values were calculated using a two‐tailed *t*‐test.

### 4.17 PROTEOSTAT analysis

The PROTEOSTAT protein aggregation assay kit provided by Enzo Life Sciences, Inc. (Miyake et al., 2022) was employed to monitor the total quantity of protein aggregation in *C. elegans* samples. This assay kit can sensitively detect various protein aggregates in solutions and is utilized to evaluate the rate of protein aggregation in samples as they age. In the detailed procedure, samples were first diluted in phosphate‐buffered saline (PBS), and then the protein concentration was determined using the CWBIO BCA Protein Assay Kit (CW0014S). For the PROTEOSTAT assay, 98 μL of the protein of interest was added to each well, with a recommended concentration range of 1 μg/mL to 10 mg/mL. To ensure experimental accuracy, the protein from *C. elegans* was diluted to a consistent concentration within the recommended range of the assay buffer. Subsequently, the samples were transferred to a 96‐well microplate containing 2 μL of the diluted PROTEOSTAT detection reagent in each well. Finally, fluorescence was measured using a fluorescence microplate reader (BMG LABTECH; CLARIOstarplus), with an excitation wavelength of 550 nm and an emission wavelength of 600 nm.

### 4.18 Western blot analysis

A previously reported standard method was utilized to analyze ubiquitinated proteins and A‐beta proteins using Western blotting (Koyuncu et al., 2021). Nematodes were lysed in a lysis buffer containing 50 mM Tris–HCl (pH 7.8), 150 mM NaCl, 0.25% sodium deoxycholate, 1 mM EDTA, 25 mM N‐ethylmaleimide, 2 mM sodium orthovanadate, 1 mM PMSF, and a protease inhibitor cocktail (MCE) using an ultrasonic cell disruptor (SCIENTZ‐650E). Subsequently, the nematode lysates were centrifuged at 12000×*g* for 10 min at 4°C, and the supernatant was collected. Protein concentration was determined using the CWBIO BCA protein assay kit (CW0014S). Based on the supernatant volume, an appropriate amount of 5× loading buffer was added, and the mixture was boiled at 100°C for 10 min. Afterwards, 20 μg of total protein was separated by SDS‐PAGE, transferred to a polyvinylidene difluoride membrane (millipore) for immunoblotting. Western blot analysis was performed using the following antibodies: anti‐ubiquitin linkage‐specific K48 antibody (Abcam, EP8589, 1:200, ab140601), goat anti‐rabbit IgG (H+L) HRP‐conjugated antibody (Elabscience, 1:1000, E‐AB‐1003), β‐amyloid 1–16 (6E10) (1:1000, PTM‐20007; PTM Bio), and beta tubulin antibody.

### 4.19 Alpha‐synuclein inclusion measurement

Continue with the previous method (van Ham et al., 2008). To quantify the number of inclusion bodies, we analyzed all foci from the nasal region to the tonsillar area. Using ImageJ software, we measured the inclusion bodies.

### 4.20 Motility

A previously reported thrashing assay was used to analyze the motility of the worms (Volovik et al., 2014). The specific procedure involved transferring a single worm expressing Alpha‐synuclein (genotype pkIs2386) on the fifth day to a droplet of M9 placed on a coverslip, and then recording the number of body bends every 30 s under a stereoscope.

### 4.21 Statistical analysis

All graphed data are presented as the mean ± SEM/SD from at least three biological replicate experiments performed in triplicate technical repeats. The RNA‐seq data were analyzed using the Ballgown package of the R project and submitted to GEO (GSE262666). Statistical calculations were performed using GraphPad Prism 5 (GraphPad Software) and SPSS (IBM Statistics 21). The mean fluorescence intensity and Western blot bands were quantified using ImageJ. Lifespan curves were generated using the Kaplan–Meier method, and differences in the overall survival rate were determined using the log‐rank test. Relative expression levels of genes were determined using the 2^−ΔΔCT^ method. The frequency distribution of a categorical variable within two groups was tested using the Chi‐square test. Differences between two groups were analyzed using two‐tailed Student's *t*‐test, with a *p* < 0.05 considered statistically significant. Statistical significance values are indicated as follows: *p < 0.05; **p < 0.01; ***p < 0.001; and n.s., not significant. Further details are provided in the figure legends.

## AUTHOR CONTRIBUTIONS

Z.Q., X.Y., F.Z.D., Y.T.Z, and D.Y.R performed experiments. S.Q.Z., Z.Q. and X.Y. analyzed data. S.Q.Z. and Z.Q. designed and supervised the entire project and prepared the manuscript.

## CONFLICT OF INTEREST STATEMENT

All authors declare no conflict of interest.

## Supporting information


Data S1.

## Data Availability

All data are available in the main text and the supporting information. Source data are provided with this paper. RNA sequencing data that support the findings of this study have been deposited in GEO with the accession code: GSE262666.
