# Supplementary material for: Cysteine protease cathepsin B promotes lysosome integrity to extend the lifespan of alternative day fasting worms
Source: Aging Cell. 2024 Jul 24;23(11):e14286. doi: 10.1111/acel.14286 (PMC11561666; doi:10.1111/acel.14286)
Supplement: Supplementary file 1 — Data S1. [file ACEL-23-e14286-s001.pdf]

Supporting information

Figure S1. ADF causes developmental damage in young adult worms.

(a) ADF reduces embryo developmental time and induces early hatching. At least 10 eggs were traced in each treatment. Data show mean time±SD. (b) Time lapses of the first 30 eggs laid by each worm were traced. 15 worms were traced for each treatment. Data show mean number ± SD. (c) ADF causes early hatching of eggs inside young adult worms. Bag worms were calculated. 15 worms were traced for each treatment. Data show mean number±SD. (a-c) N2 worms were used. ***: $P<0.001$, t-test. Ctrl: Control worms. (d) TGF-beta and WNT signaling pathways were significantly affected by ADF in young stage worms according to RNA-seq. (e) TGF-beta and WNT regulated genes were significantly reduced by ADF. Heatmap: genes significantly changed (>1.5 fold). (f) Real-time PCR confirmed downregulation of *cyd-1* and *cfz-2* by ADF. ***: $P<0.001$, t-test. Ctrl: Control housekeeping gene. (g-h) ADF failed to further reduce lifespan of *cyd-1* and *cfz-2* mutants. Each survival curve is a representative of at least three independent repeats. (i) Summary of mean lifespans. Ctrl: Control worms. P values were determined using the log-rank test. ***: $P<0.001$. n.s.: no significant difference. The detailed data of lifespan was summarized in Table S2 in supporting information.

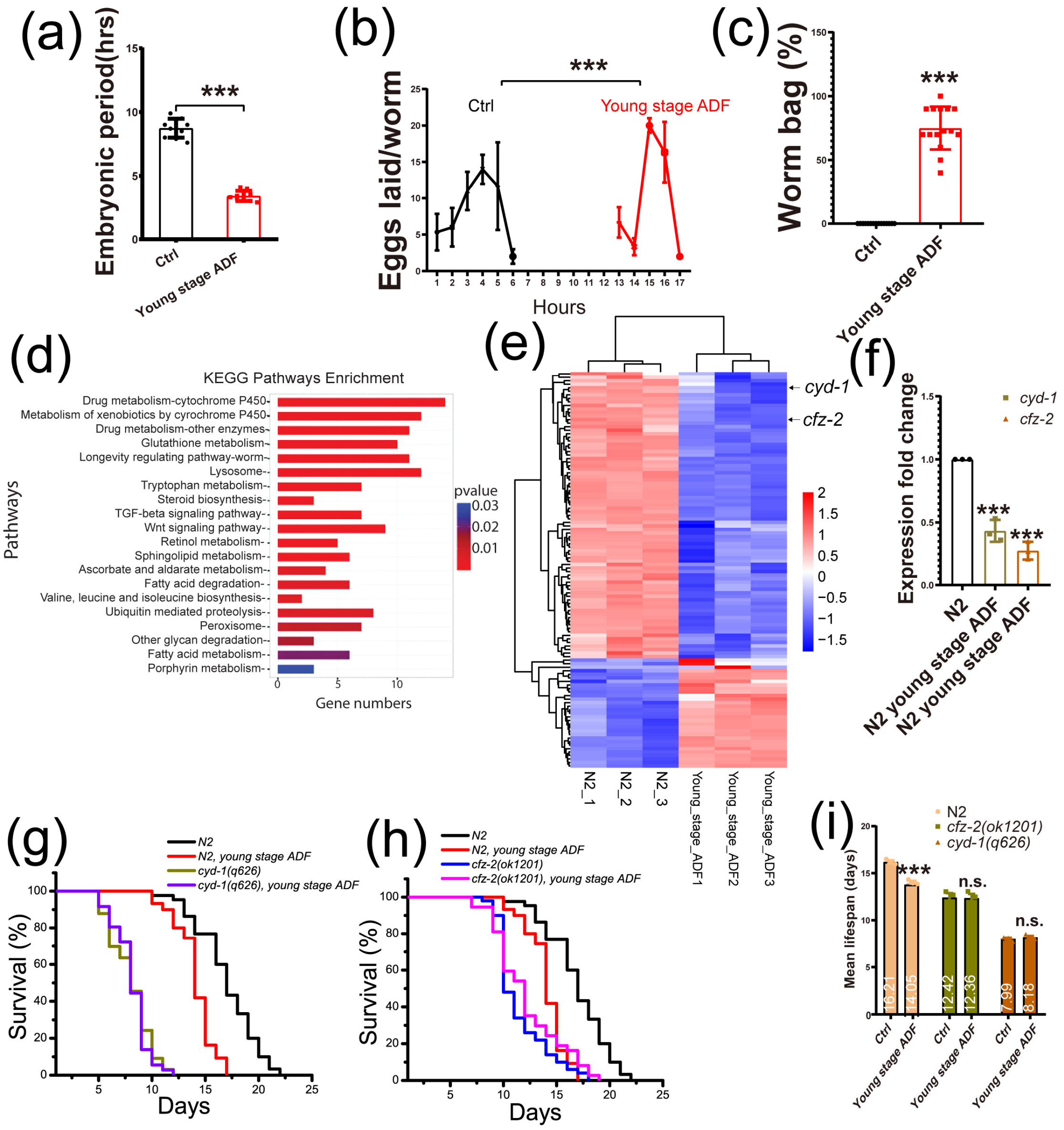
(a)
Embryonic period(hrs)
***
Ctrl
Young stage ADF
(b)
Eggs laid/worm
***
Ctrl
Young stage ADF
Hours
(c)
Worm bag (%)
***
Ctrl
Young stage ADF
(d)
KEGG Pathways Enrichment
Pathways
Drug metabolism-cytochrome P450
Metabolism of xenobiotics by cyrochrome P450
Drug metabolism-other enzymes
Glutathione metabolism
Longevity regulating pathway-worm
Lysosome
Tryptophan metabolism
Steroid biosynthesis
TGF-beta signaling pathway
Wnt signaling pathway
Retinol metabolism
Sphingolipid metabolism
Ascorbate and aldarate metabolism
Fatty acid degradation
Valine, leucine and isoleucine biosynthesis
Ubiquitin mediated proteolysis
Peroxisome
Other glycan degradation
Fatty acid metabolism
Porphyrin metabolism
pvalue
Gene numbers
(e)
cyd-1
cfz-2
N2_1
N2_2
N2_3
Young_stage_ADF1
Young_stage_ADF2
Young_stage_ADF3
(f)
cyd-1
cfz-2
Expression fold change
***
***
N2
N2 young stage ADF
N2 young stage ADF
(g)
N2
N2, young stage ADF
cyd-1(q626)
cyd-1(q626), young stage ADF
Survival (%)
Days
(h)
N2
N2, young stage ADF
cfz-2(ok1201)
cfz-2(ok1201), young stage ADF
Survival (%)
Days
(i)
N2
cfz-2(ok1201)
cyd-1(q626)
Mean lifespan (days)
***
n.s.
n.s.
16.21
14.05
12.42
12.36
7.99
8.18
Ctrl
Young stage ADF
Ctrl
Young stage ADF
Ctrl
Young stage ADF

Figure S2. ADF extends lifespan through *cpr-2/5* in different tissues.

(a) Lifespan affected by ADF performed on midlife age worms in *cpr-2/5* tissue-specific knocking down. Ctrl: RNAi control strain L4440. (c) Tissue-specific overexpression of *cpr-2* can promote the lifespan of wild-type worms. Ctrl: transgenic injection strains with empty expression vector L2528. Each survival curve is representative of at least three independent repeats. (b, d) Summary of mean lifespan. P values were determined using the log-rank test. ***: P<0.001. The detailed data of lifespan was summarized in Table S2 in supporting information.

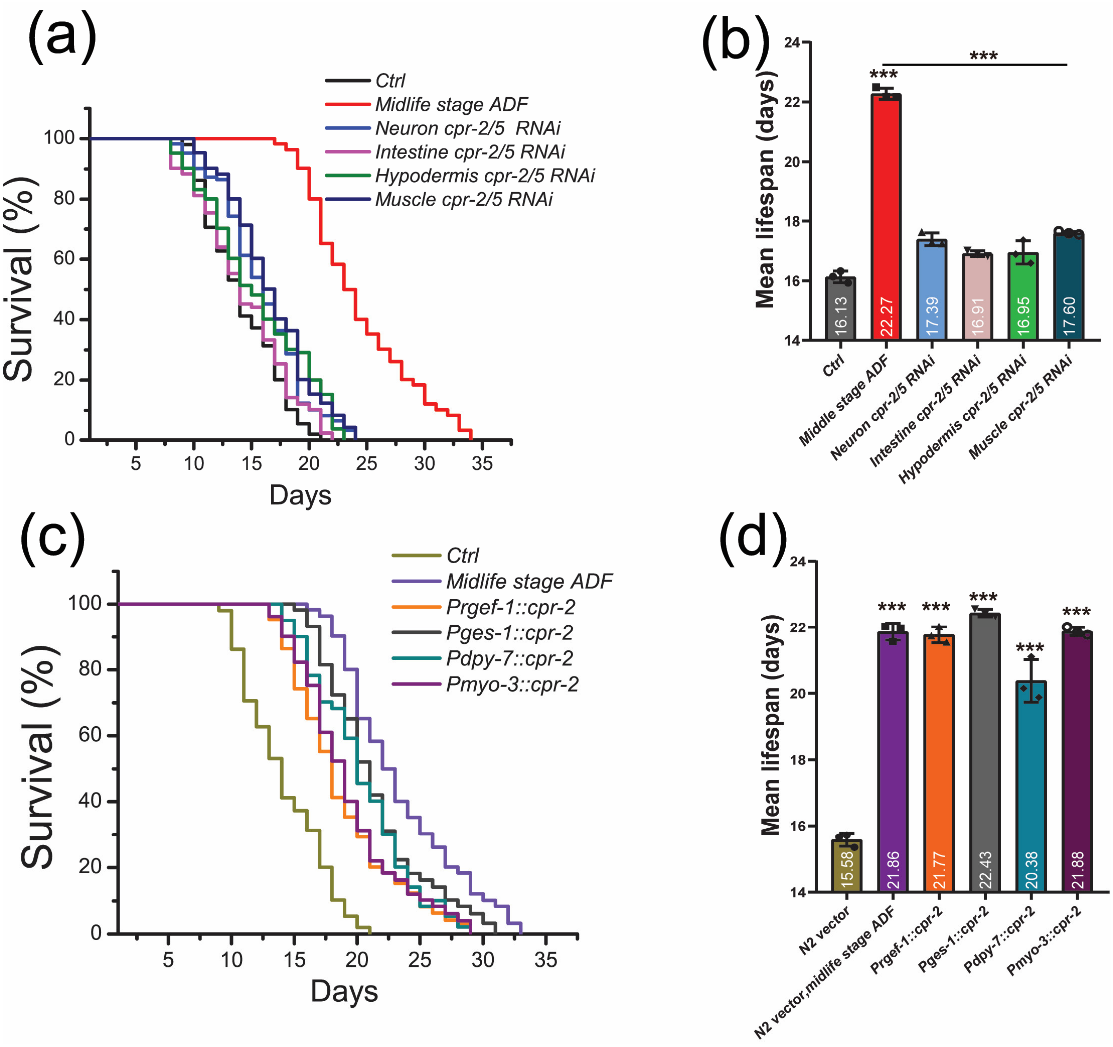

Figure S3. ADF performed on midlife age worms provided with animal-based protein can maintain lysosome morphology in the body and intestine.

(a-b) Confocal fluorescence images of the body wall muscles in control worms, *cpr-2/5* RNAi, and ADF worms expressing LAAT-1::GFP. (c-d) Confocal fluorescence images of the intestines in control worms, *cpr-2/5* RNAi, and ADF worms expressing LAAT-1::GFP. White arrow indicates vesicular lysosomes. (e-h) The number of vesicular lysosomes was quantified. At least 10 animals were scored in each treatment for each repeat. Data are shown as mean±SD. P values were calculated using t-test. *: P<0.05, **: P<0.01, ***: P<0.001. n.s., no significance. Scale bars: 25 μm. E-64: 20 μg/mL.

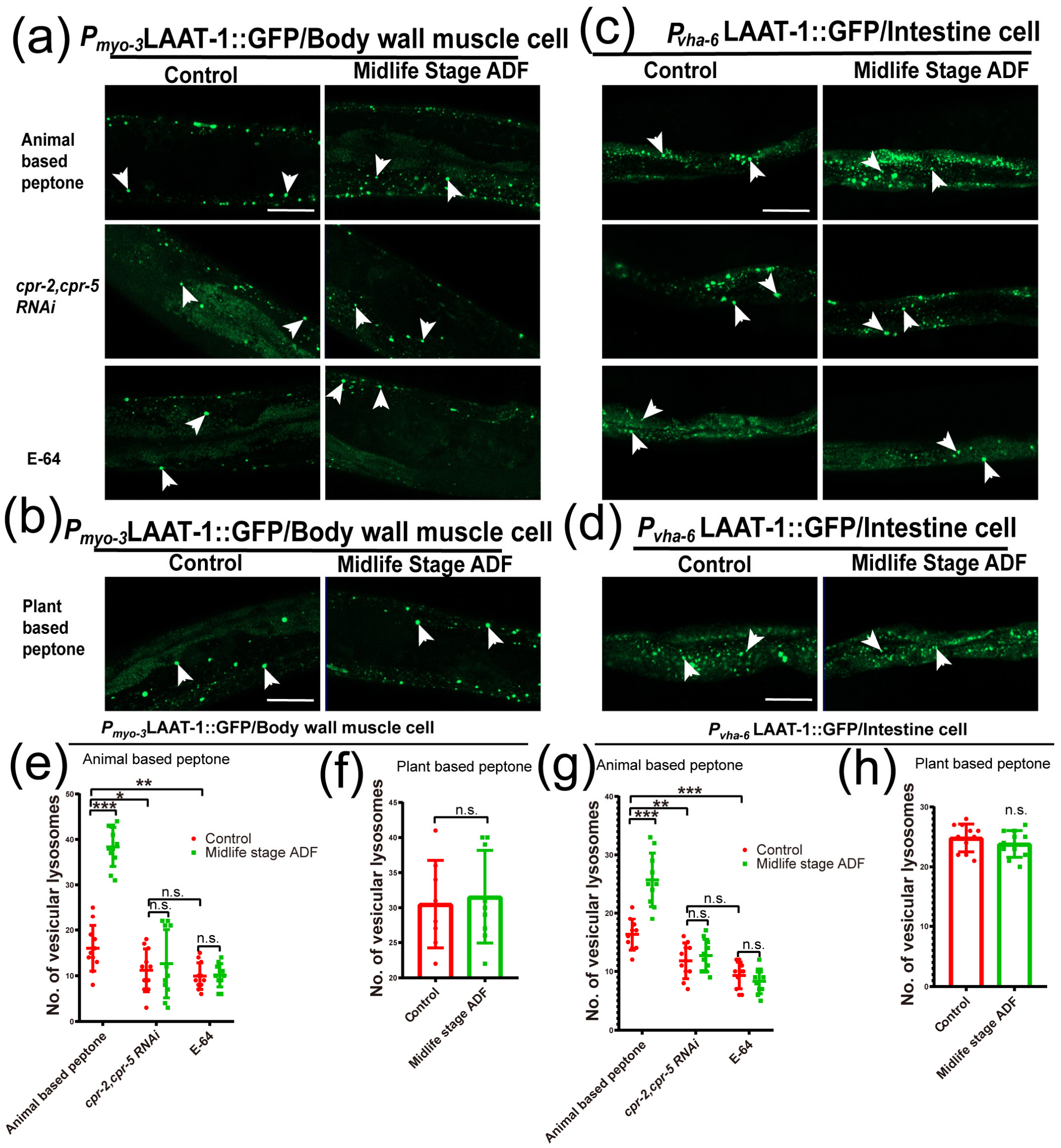

Figure S4. ADF performed on midlife age worms extends lifespan through IIS, *eat-2*, and *skn-1*. The lifespan of *eat-2* (a), *daf-2* (b), *daf-16* (c), and *skn-1* (d) is affected by ADF performed on midlife age worms. Each survival curve is representative of at least three independent repeats. (e) Summary of lifespan data. P values were determined using the log-rank test. n.s.: no significant difference. ***: P<0.001. The detailed data of lifespan was summarized in Table S3 in supporting information. (f) ADF enhances DAF-16 nuclear translocation and measurements of DAF-16 nuclear localization. Predominate: Nuclear translocation in cells throughout the body, partial nuclear: some cells show nuclear translocation, cytoplasmic: no nuclear translocation. Sample size was more than 30 in each treatment. P<0.001, Chi-square. (g) The mean intensity of GST-4::GFP, the reporter of SKN-1, is affected by ADF performed on midlife age worms. Mean intensity±SEM is shown below relative to control worms treated in parallel in 3 independent experiments. At least 10 worms were tested in each repeat. ***: P<0.001. t-test. Scale bars: 100 µm.

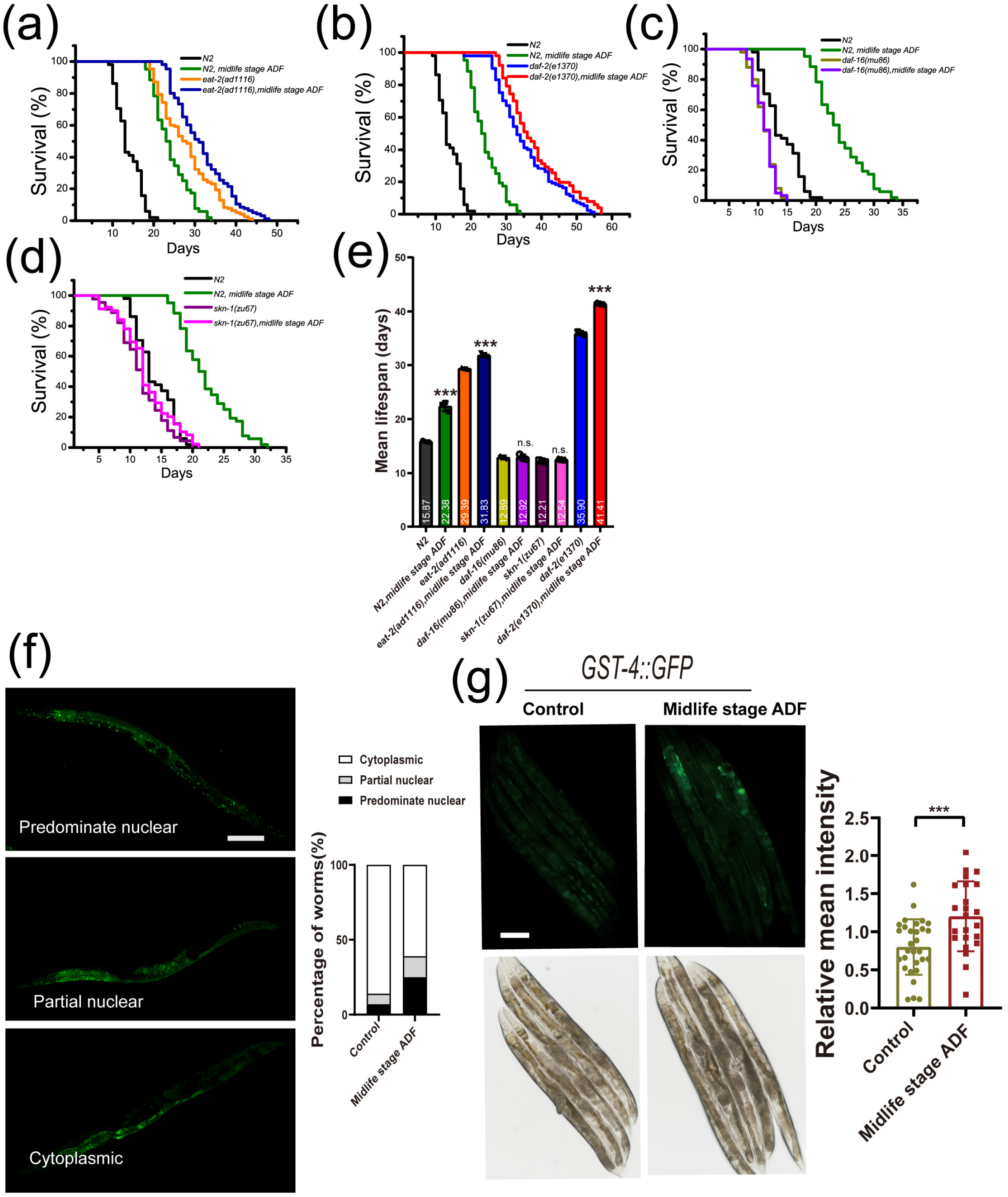
(a)
(b)
(c)
(d)
(e)
(f)
(g)
Survival (%)
Days
N2
N2, midlife stage ADF
eat-2(ad1116)
eat-2(ad1116),midlife stage ADF
daf-2(e1370)
daf-2(e1370),midlife stage ADF
daf-16(mu86)
daf-16(mu86),midlife stage ADF
skn-1(zu67)
skn-1(zu67),midlife stage ADF
Mean lifespan (days)
15.87
22.38
29.39
31.83
12.89
12.92
12.21
12.54
35.90
41.41
n.s.
Predominate nuclear
Partial nuclear
Cytoplasmic
Percentage of worms(%)
Control
Midlife stage ADF
GST-4::GFP
Relative mean intensity

Figure S5. ADF effects depend on lysosome biogenesis and lipophagy, but not autophagy.

(a) *hlh-30* mutants reduced the lifespan extension induced by ADF in midlife age worms. Each survival curve is representative of at least three independent repeats. (b) Summary of the lifespan data. P values were determined using the log-rank test. ***: P<0.001. The detailed data of lifespan was summarized in Table S3 in supporting information. (c) ADF performed on midlife age worms enhances the nuclear translocation of HLH-30::GFP. Predominate: Nuclear translocation in cells throughout the body; partial nuclear: some cells show nuclear translocation; cytoplasmic: no nuclear translocation. Sample size was more than 30 in each treatment. P<0.001, Chi-square test. The scale bar represents 100 μm. (d) Knocking down *let-363*, an ortholog of human mTOR, partially reduced the lifespan extension induced by ADF in midlife age worms. Each survival curve is representative of at least three independent repeats. (e-f) Confocal images of pLGG-1::GFP and pSQST-1::GFP in control and ADF worms. pLGG-1::GFP and pSQST-1::GFP puncta correspond to autophagosomes. The non-conjugated form of LGG-1 and LGG-2 presents a diffuse localization pattern. Sample size was more than 10 in each treatment for each repeat. n.s.: no significant difference, as determined by t-test. The scale bar represents 25 μm. (g) The expression of *lipl-3* is affected by ADF in midlife age worms, as tested by real-time PCR. ***: P<0.001, as determined by t-test. (h) RNAi of *lipl-3* reduced the lifespan extension induced by ADF in midlife age worms. Ctrl: control, worms fed with RNAi L4440 strain. Each survival curve is representative of at least three independent repeats. (i) Summary of the lifespan data. ***: P<0.001. P values were determined using the log-rank test. (j-k) Oil red O staining of control and ADF midlife age worms reveals that ADF promotes lipid metabolism. The scale bar represents 100 μm. Mean±SEM are shown below relative to control worms treated in parallel in 3 independent experiments. At least 10 worms were tested in each repeat. ***: P<0.001, as determined by t-test.

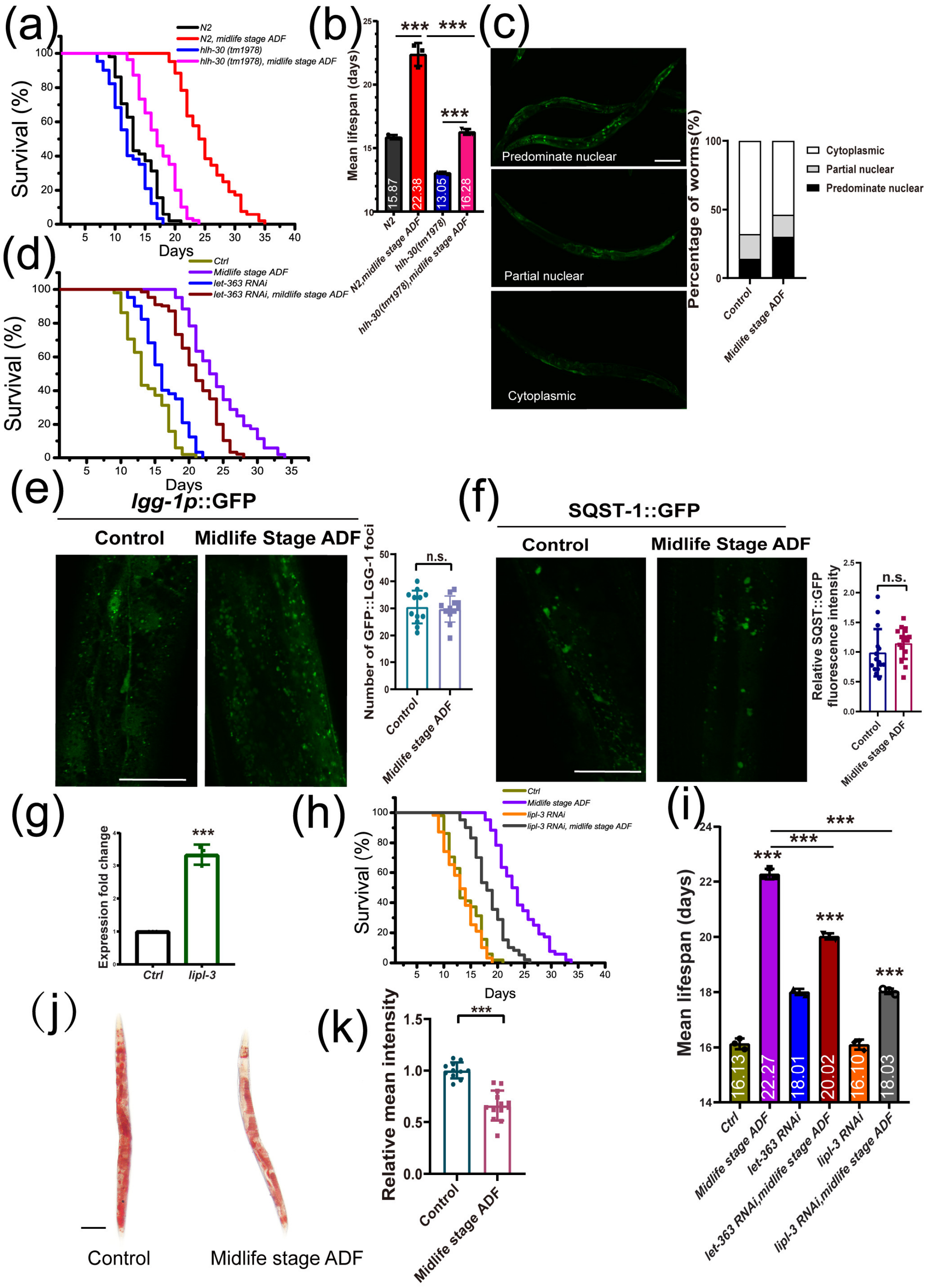
(a)
N2
N2, midlife stage ADF
hlh-30 (tm1978)
hlh-30 (tm1978), midlife stage ADF
Survival (%)
Days
(b)
Mean lifespan (days)
15.87
22.38
13.05
16.28
N2
N2,midlife stage ADF
hlh-30(tm1978)
hlh-30(tm1978),midlife stage ADF
(c)
Predominate nuclear
Partial nuclear
Cytoplasmic
Percentage of worms(%)
Cytoplasmic
Partial nuclear
Predominate nuclear
Control
Midlife stage ADF
(d)
Ctrl
Midlife stage ADF
let-363 RNAi
let-363 RNAi, midlife stage ADF
Survival (%)
Days
(e)
lgg-1p::GFP
Control
Midlife Stage ADF
Number of GFP::LGG-1 foci
n.s.
Control
Midlife stage ADF
(f)
SQST-1::GFP
Control
Midlife Stage ADF
Relative SQST::GFP fluorescence intensity
n.s.
Control
Midlife stage ADF
(g)
Expression fold change
Ctrl
lipl-3
(h)
Ctrl
Midlife stage ADF
lipl-3 RNAi
lipl-3 RNAi, midlife stage ADF
Survival (%)
Days
(i)
Mean lifespan (days)
16.13
22.27
18.01
20.02
16.10
18.03
Ctrl
Midlife stage ADF
let-363 RNAi
let-363 RNAi,midlife stage ADF
lipl-3 RNAi
lipl-3 RNAi,midlife stage ADF
(j)
Control
Midlife stage ADF
(k)
Relative mean intensity
Control
Midlife stage ADF

Figure S6. Midlife stage ADF reduces alpha-synuclein accumulation.

(a) Alpha-synuclein-YFP expression in the head region and the average number of inclusions per animal between the tip of the nose and the pharyngeal bulb of control and ADF *pkIs2386* [*unc-54p::α-syn::YFP*] worms. (b-d) Mean intensity of *α-syn*::YFP. Relative mean intensity±SEM is shown below relative to control worms treated in parallel in 3 independent experiments. (e-g) Average of inclusions per animal. Data show the mean number±SD. **: P<0.01, ***: P<0.001, n.s.: no significant difference. At least 10 worms were tested in each repeat. Scale bar: 25 μm. E-64: 20 μg/mL.

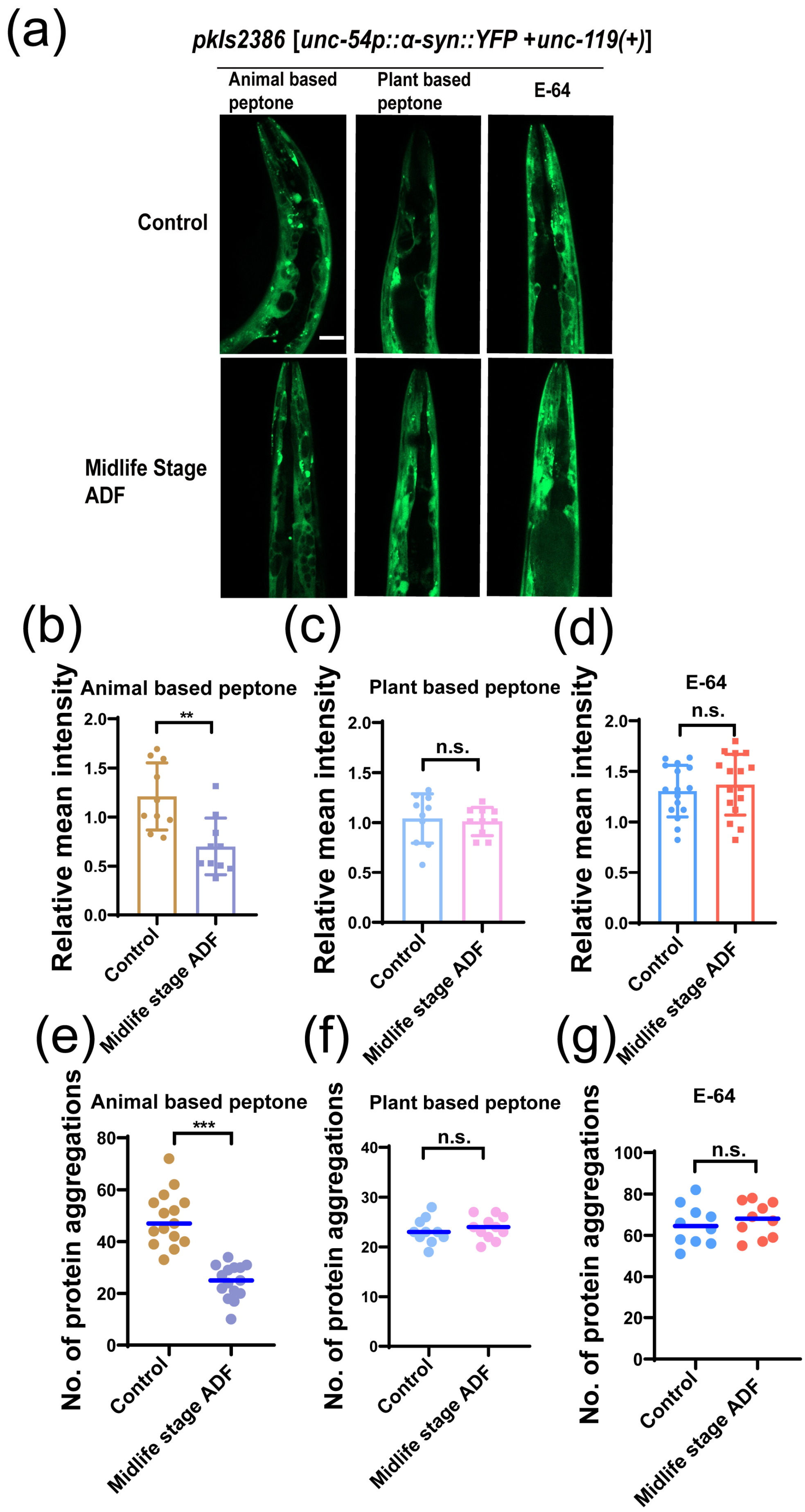
(a)
pkIs2386 [unc-54p::α-syn::YFP +unc-119(+)]
Animal based peptone
Plant based peptone
E-64
Control
Midlife Stage ADF
(b)
Animal based peptone
Relative mean intensity
**
Control
Midlife stage ADF
(c)
Plant based peptone
Relative mean intensity
n.s.
Control
Midlife stage ADF
(d)
E-64
Relative mean intensity
n.s.
Control
Midlife stage ADF
(e)
Animal based peptone
No. of protein aggregations
***
Control
Midlife stage ADF
(f)
Plant based peptone
No. of protein aggregations
n.s.
Control
Midlife stage ADF
(g)
E-64
No. of protein aggregations
n.s.
Control
Midlife stage ADF

Figure S7. Midlife stage ADF enhances dopaminergic neuron integrity.

(a) DAT-1::GFP expression in the head region of control and ADF worms. (b-d) The relative mean intensity of DAT-1::GFP. ***: P<0.001, n.s.: no significant difference. Scale bar: 25 μm. Mean fold± SEM is shown below relative to control worms treated in parallel in 3 independent experiments. At least 10 worms were tested in each repeat. (e) The mobility of control and ADF midlife age worms assessed using thrashing experiments. Animals that were alive but unable to swim were scored as paralyzed. Motility assays were carried out by counting body bends over a 30-second interval in M9 at day 5 adulthood. **: P<0.01, n.s.: no significant difference. P values were determined using two-sided t-test. Mean±SD is shown below relative to control worms treated in parallel in 3 independent experiments. At least 10 worms were tested in each repeat. E-64: 20 μg/mL.

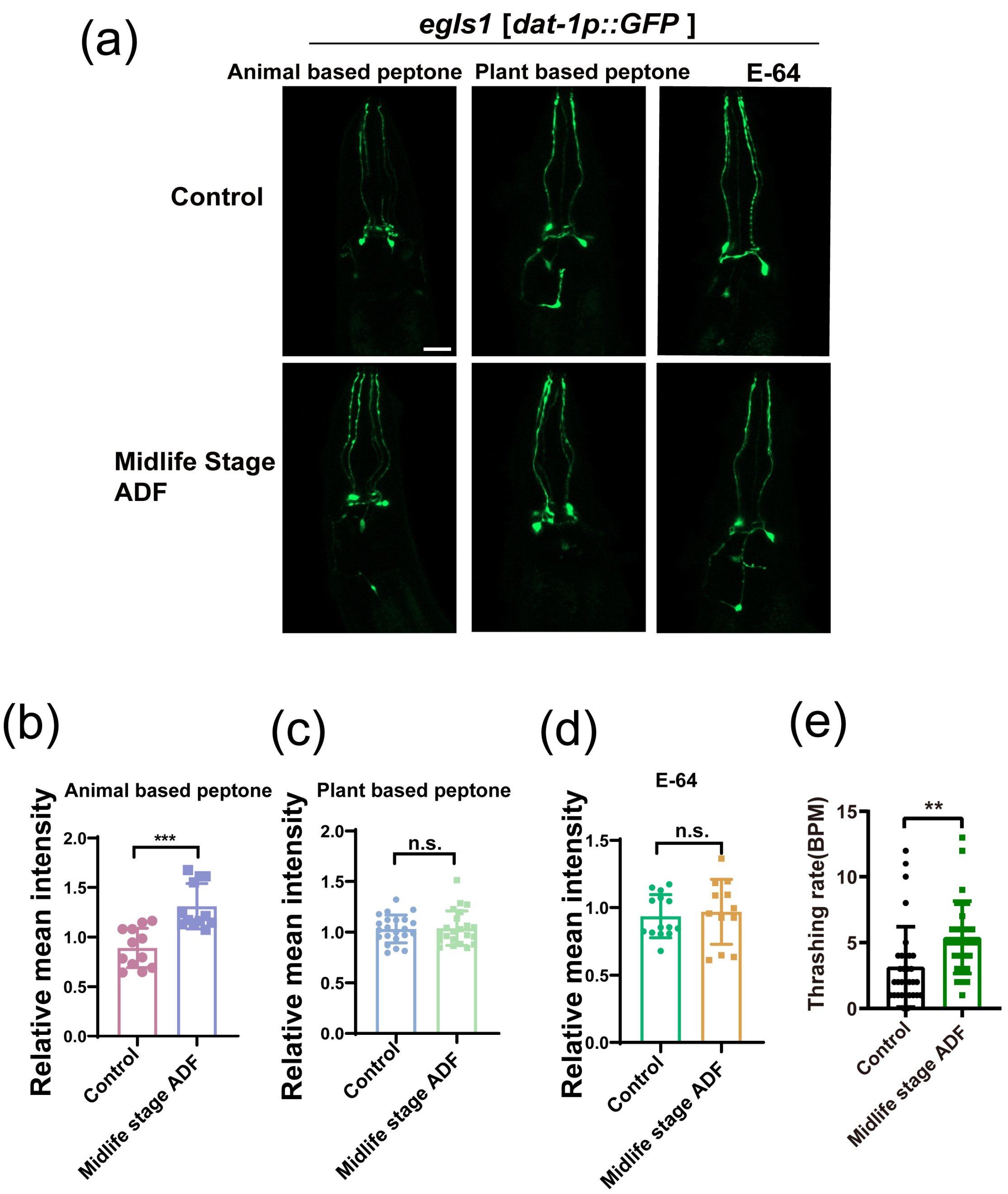

Figure S8. Midlife stage ADF reduces toxic protein aggregation.

(a) Western blots of A-beta aggregation in control and ADF midlife age *dvIs2* [*Punc-54::human A-beta 3-42; pRF4 (rol-6(su1006)*] worms. (b) The relative fold changes of WB. n.s.: no significant difference. *: P<0.05, ***: P<0.001. P values were determined using two-sided t-test. (c) Percentage of paralysis control and ADF midlife age *dvIs2* [*Punc-54::human A-beta 3-42; pRF4 (rol-6(su1006)*] worms **: P<0.01. P values were determined using two-sided t-test. Mean±SD is shown below relative to control worms treated in parallel in 3 independent experiments. At least 10 worms were tested in each repeat. E-64: 20 μg/mL.

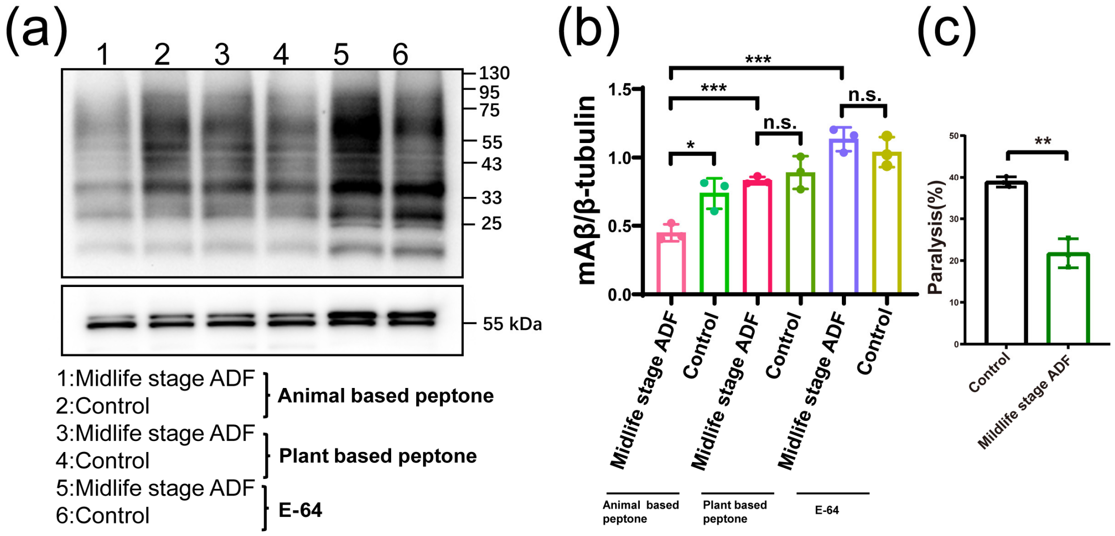

Figure S9. The expression of *cpr-2/5*, *cyd-1* and *cfz-2* in young and midlife stage ADF worms.

(a) The expression of *cpr2/5*, *cyd-1* and *cfz-2* in worms cultured on plant-based peptone treated with young stage ADF. (b) The expression of *cpr2/5*, *cyd-1* and *cfz-2* in worms cultured on plant-based peptone treated with midlife stage ADF. (c) The expression of *cyd-1* and *cfz-2* in worms cultured on animal-based peptone treated with midlife stage ADF. (d) The expression of *cpr2/5* in worms cultured on animal-based peptone treated with young stage ADF. n.s.: no significant difference. ***: P<0.001. P values were determined using two-sided t-test.

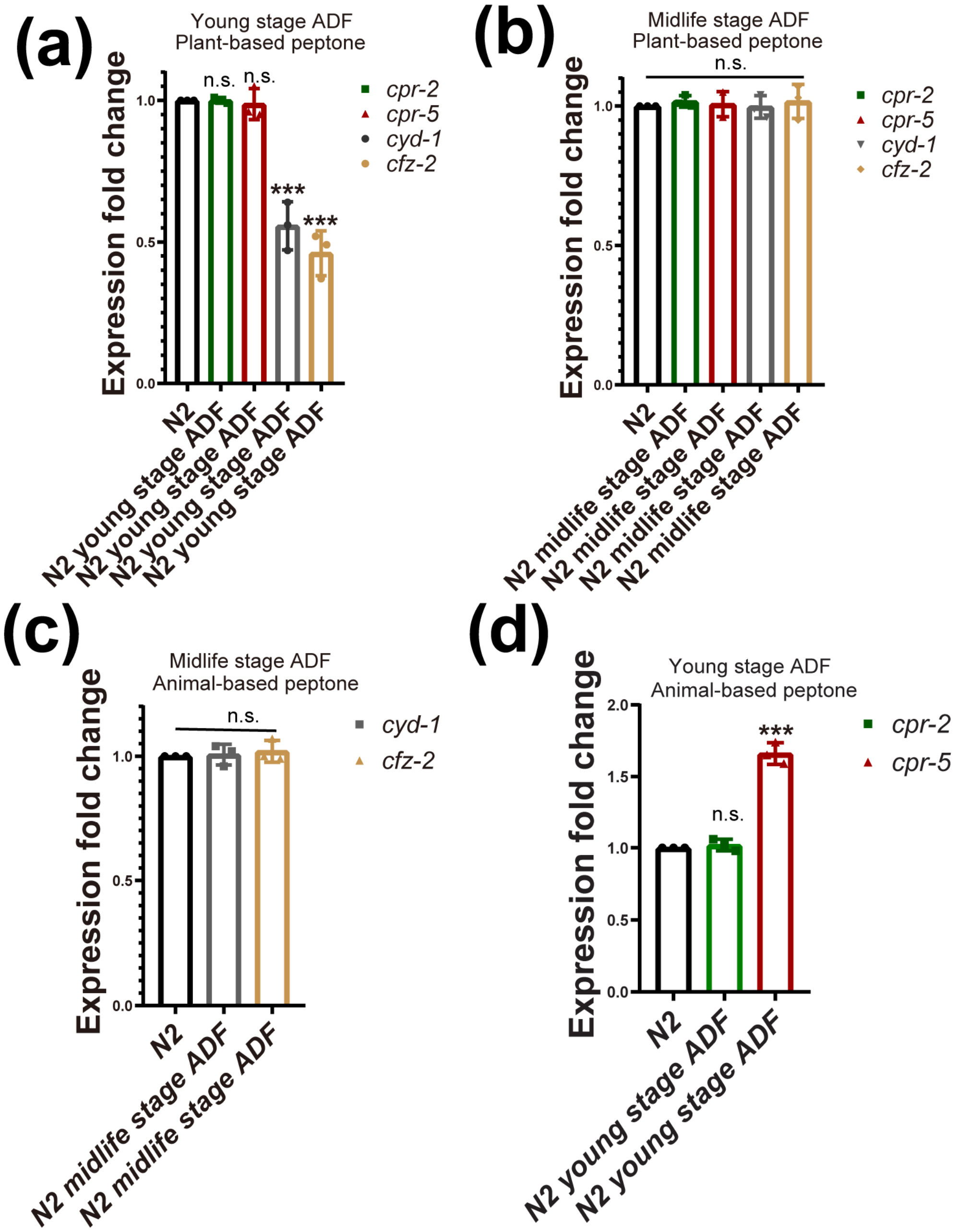

Table S1. The lifespan of worms treated with ADF.

Strains	Protein source	treatment	group	Mean life Span ±SEM(Days)	Maximum life span (Days)	P value	N
N2	animal-based peptone	Control	Exp 1	15.50±0.48	23	/	76
			Exp 2	16.06±0.39	22	/	68
			Exp 3	15.76±0.56	22	/	73
		Young Stage ADF	Exp 1	13.95±0.20	17	<0.001	81
			Exp 2	13.86±0.20	18	<0.001	61
			Exp 3	14.33±0.21	17	<0.001	75
		Midlife Stage ADF	Exp 1	21.44±0.53	31	<0.001	65
			Exp 2	23.40±0.60	33	<0.001	66
			Exp 3	22.90±0.69	34	<0.001	69
		Control	Exp 1	19.23±0.39	23	/	76
			Exp 2	19.63±0.56	24	/	68
			Exp 3	19.52±0.22	24	/	73
		Senile Stage ADF	Exp 1	20.15±0.22	25	n.s.	75
			Exp 2	19.58±0.14	24	n.s.	67
			Exp 3	19.67±0.26	25	n.s.	70
	plant-based peptone	Control	Exp 1	17.83±0.42	25	/	66
			Exp 2	18.20±0.15	25	/	78
			Exp 3	18.53±0.62	25	/	63
		Young Stage ADF	Exp 1	14.35±0.22	17	<0.001	61
			Exp 2	14.63±0.15	16	<0.001	71
			Exp 3	14.39±0.08	16	<0.001	65
		Midlife Stage ADF	Exp 1	17.29±0.30	25	n.s.	61
			Exp 2	17.69±0.43	25	n.s.	65
			Exp 3	17.96±0.45	24	n.s.	73
		Control	Exp 1	20.85±0.22	25	/	63
			Exp 2	20.93±0.35	25	/	60
			Exp 3	21.06±0.09	25	/	62
		Senile Stage ADF	Exp 1	21.04±0.28	25	n.s.	65
			Exp 2	20.18±0.33	25	n.s.	67
			Exp 3	20.69±0.12	25	n.s.	70
fem-3 (e2006)	animal-based peptone	Control	Exp 1	13.54±0.11	20	/	60
			Exp 2	13.06±0.08	21	/	63
			Exp 3	13.26±0.16	20	/	71
		Young Stage ADF	Exp 1	11.25±0.18	15	<0.001	66
			Exp 2	10.59±0.54	14	<0.001	62
			Exp 3	11.06±0.31	15	<0.001	70
	plant-based peptone	Control	Exp 1	13.95±0.22	21	/	62
			Exp 2	13.88±0.12	21	/	67
			Exp 3	14.01±0.59	22	/	61

		Young Stage ADF	Exp 1	11.46±0.32	15	<0.001	60
			Exp 2	11.51±0.17	16	<0.001	74
			Exp 3	11.36±0.19	16	<0.001	63

Mean lifespans were calculated by using Kaplan-Meier method; P values were determined using the log-rank test; SEM: standard error of the mean; N: number of worms used. n.s.: no significant difference.

Table S2. The lifespan data of worms affected by ADF, *cpr-2*, *cpr-5*, *cfz-2* and *cyd-1*.

Strains	treatment	group	Mean life Span ±SEM(Days)	Maximum life span(Days)	P value	N
N2	Control	Exp 1	16.50±0.44	23	/	66
		Exp 2	16.18±0.33	22	/	68
		Exp 3	15.96±0.18	22	/	63
	Young Stage ADF	Exp 1	13.95±0.20	17	<0.001	61
		Exp 2	13.86±0.20	18	<0.001	61
		Exp 3	14.33±0.21	17	<0.001	65
cfz-2(ok1201)	Control	Exp 1	11.52±0.35	18	/	60
		Exp 2	12.70±0.41	17	/	68
		Exp 3	13.05±0.53	18	/	66
	Young Stage ADF	Exp 1	12.49±0.51	19	n.s.	67
		Exp 2	13.07±0.66	19	n.s.	65
		Exp 3	11.52±0.53	18	n.s.	68
cyd-1(q626)	Control	Exp 1	8.15±0.33	13	/	64
		Exp 2	7.71±0.31	12	/	62
		Exp 3	8.11±0.27	12	/	67
	Young Stage ADF	Exp 1	8.00±0.42	12	n.s.	62
		Exp 2	8.50±0.37	13	n.s.	69
		Exp 3	8.03±0.34	12	n.s.	64
N2	Control	Exp 1	15.78±0.32	23	/	66
		Exp 2	16.06±0.39	22	/	68
		Exp 3	15.76±0.56	22	/	63
	Midlife Stage ADF	Exp 1	21.36±0.15	31	<0.001	65
		Exp 2	22.69±0.62	33	<0.001	66
		Exp 3	23.08±0.11	34	<0.001	69
cpr-2(ok2833)	Control	Exp 1	14.65±0.43	21	/	63
		Exp 2	14.26±0.68	20	/	67
		Exp 3	13.96±0.47	20	/	65
	Midlife Stage ADF	Exp 1	14.12±0.55	22	n.s.	69
		Exp 2	13.64±0.41	21	n.s.	64
		Exp 3	13.46±0.45	20	n.s.	60
cpr-5(ok2344)	Control	Exp 1	16.06±0.44	20	/	66
		Exp 2	15.51±0.49	20	/	61
		Exp 3	16.57±0.47	21	/	66
	Midlife Stage ADF	Exp 1	17.96±0.46	25	<0.001	66
		Exp 2	17.16±0.53	25	<0.001	62
		Exp 3	18.29±0.57	26	<0.001	64
N2	Midlife Stage ADF	Exp 1	21.36±0.15	31	/	65
		Exp 2	22.69±0.62	33	/	66
		Exp 3	23.08±0.11	34	/	69

cpr-2(ok2833)		Exp 1	14.12±0.55	22	<0.001	69
		Exp 2	13.64±0.41	21	<0.001	64
		Exp 3	13.46±0.45	20	<0.001	60
cpr-5(ok2344)		Exp 1	17.96±0.46	25	<0.001	66
		Exp 2	17.16±0.53	25	<0.001	62
		Exp 3	18.29±0.57	26	<0.001	64
N2, 20μg/mL E-64		Exp 1	15.95±0.44	21	/	66
		Exp 2	16.66±0.93	21	/	64
		Exp 3	16.62±0.43	21	/	64
	Midlife Stage ADF	Exp 1	15.73±0.42	20	n.s.	61
		Exp 2	16.06±0.36	21	n.s.	63
		Exp 3	16.57±0.43	21	n.s.	60
N2	Midlife Stage ADF	Exp 1	21.36±0.15	31	/	65
		Exp 2	22.69±0.62	33	/	66
		Exp 3	23.08±0.11	34	/	69
N2, 20μg/mL E-64		Exp 1	15.73±0.42	20	<0.001	61
		Exp 2	16.06±0.36	21	<0.001	63
		Exp 3	16.57±0.43	21	<0.001	60
N2	Control(L4440)	Exp 1	15.93±0.24	20	/	65
		Exp 2	16.14±0.15	21	/	62
		Exp 3	16.32±0.21	21	/	62
	Midlife Stage ADF	Exp 1	22.16±0.46	33	<0.001	63
		Exp 2	22.49±0.34	34	<0.001	60
		Exp 3	22.17±0.52	33	<0.001	78
cpr-2/5 RNAi	Control(L4440)	Exp 1	14.39±0.21	20	/	74
		Exp 2	15.05±0.43	20	/	71
		Exp 3	15.26±0.07	21	/	68
	Midlife Stage ADF	Exp 1	15.16±0.47	21	n.s.	63
		Exp 2	15.46±0.11	20	n.s.	62
		Exp 3	15.37±0.76	20	n.s.	69
N2	Midlife Stage ADF	Exp 1	22.16±0.46	33	/	63
		Exp 2	22.49±0.34	34	/	60
		Exp 3	22.17±0.52	33	/	78
cpr-2/5 RNAi		Exp 1	15.16±0.47	21	<0.001	63
		Exp 2	15.46±0.11	20	<0.001	62
		Exp 3	15.37±0.76	20	<0.001	69
N2 vector		Exp 1	15.35±0.41	21	/	68
		Exp 2	15.66±0.07	20	/	66
		Exp 3	15.72±0.18	21	/	68
	Midlife Stage ADF	Exp 1	21.98±0.76	32	<0.001	92
		Exp 2	22.03±0.16	33	<0.001	60
		Exp 3	21.57±0.71	32	<0.001	65

cpr-2 oe		Exp 1	22.16±0.11	31	/	66
		Exp 2	22.56±0.15	32	/	64
		Exp 3	22.72±0.75	32	/	71
	Midlife Stage ADF	Exp 1	21.56±0.16	32	n.s.	70
		Exp 2	22.53±0.35	33	n.s.	72
		Exp 3	22.18±0.14	33	n.s.	65
cpr-5 oe		Exp 1	18.16±0.11	26	/	89
		Exp 2	18.56±0.15	26	/	65
		Exp 3	18.72±0.75	27	/	65
	Midlife Stage ADF	Exp 1	21.05±0.26	30	<0.001	74
		Exp 2	22.13±0.15	31	<0.001	71
		Exp 3	22.47±0.12	33	<0.001	75
cpr-2/5 oe		Exp 1	23.11±0.08	34	/	82
		Exp 2	23.56±0.45	34	/	69
		Exp 3	23.72±0.18	34	/	60
	Midlife Stage ADF	Exp 1	24.15±0.04	35	n.s.	62
		Exp 2	22.17±0.88	33	n.s.	60
		Exp 3	23.56±0.74	34	n.s.	88
N2 vector		Exp 1	15.35±0.41	21	/	68
		Exp 2	15.66±0.07	20	/	66
		Exp 3	15.72±0.18	21	/	68
cpr-2 oe		Exp 1	22.16±0.11	31	<0.001	66
		Exp 2	22.56±0.15	32	<0.001	64
		Exp 3	22.72±0.75	32	<0.001	71
cpr-5 oe		Exp 1	18.16±0.11	26	<0.001	89
		Exp 2	18.56±0.15	26	<0.001	65
		Exp 3	18.72±0.75	27	<0.001	65
cpr-2/5 oe		Exp 1	23.11±0.08	34	<0.001	82
		Exp 2	23.56±0.45	34	<0.001	69
		Exp 3	23.72±0.18	34	<0.001	60
N2	Control (L4440)	Exp 1	15.93±0.24	20	/	65
		Exp 2	16.14±0.15	21	/	62
		Exp 3	16.32±0.21	21	/	62
	Midlife Stage ADF	Exp 1	22.16±0.46	33	<0.001	63
		Exp 2	22.49±0.34	34	<0.001	60
		Exp 3	22.17±0.52	33	<0.001	78
	Midlife Stage ADF	Exp 1	22.16±0.46	33	/	63
		Exp 2	22.49±0.34	34	/	60
		Exp 3	22.17±0.52	33	/	78
	neuron *cpr-2/5* RNAi	Exp 1	17.25±0.08	24	<0.001	74
		Exp 2	17.63±0.33	24	<0.001	68
		Exp 3	17.29±0.54	24	<0.001	63

	intestine *cpr-2/5* RNAi	Exp 1	16.89±0.19	22	<0.001	62
		Exp 2	16.83±0.22	22	<0.001	61
		Exp 3	17.01±0.71	22	<0.001	99
	hypodermis *cpr-2/5* RNAi	Exp 1	17.36±0.35	23	<0.001	88
		Exp 2	16.59±0.21	23	<0.001	85
		Exp 3	16.89±0.13	23	<0.001	74
	muscle *cpr-2/5* RNAi	Exp 1	17.66±0.05	24	<0.001	95
		Exp 2	17.59±0.16	24	<0.001	98
		Exp 3	17.55±0.24	24	<0.001	94
N2	Control (vector)	Exp 1	15.35±0.41	21	/	68
		Exp 2	15.66±0.07	20	/	66
		Exp 3	15.72±0.18	21	/	68
	Midlife Stage ADF	Exp 1	21.98±0.76	32	<0.001	92
		Exp 2	22.03±0.16	33	<0.001	60
		Exp 3	21.57±0.71	32	<0.001	65
	Prgef-1::cpr-2	Exp 1	21.55±0.06	29	<0.001	74
		Exp 2	21.74±0.55	29	<0.001	65
		Exp 3	22.03±0.25	29	<0.001	65
	Pges-1::cpr-2	Exp 1	22.56±0.13	31	<0.001	67
		Exp 2	22.33±0.11	31	<0.001	82
		Exp 3	22.41±0.78	31	<0.001	67
	Pdpy-7::cpr-2	Exp 1	20.15±0.09	29	<0.001	68
		Exp 2	19.88±0.18	29	<0.001	65
		Exp 3	21.11±0.25	29	<0.001	63
	Pmyo-3::cpr-2	Exp 1	22.01±0.81	29	<0.001	71
		Exp 2	21.77±0.16	29	<0.001	74
		Exp 3	21.85±0.29	29	<0.001	70

Control for RNAi: Feeding RNAi control clones with empty vector L4440. Control for overexpression: the transgenic injection strains with empty expression vector L2528. Mean lifespans were calculated by using Kaplan-Meier method; P values were determined using the log-rank test; SEM: standard error of the mean; N: number of worms used. n.s.: no significant difference. oe: overexpression expression.

Table S3. The lifespan of ADF worms affected by different signaling pathway.

Animal- based peptone						
Strains	treatment	group	Mean life Span ±SEM(Days)	Maximum life span(Days)	P value	N
N2	Control	Exp 1	15.78±0.32	23	/	66
		Exp 2	16.06±0.39	22	/	68
		Exp 3	15.76±0.56	22	/	63
	Midlife Stage ADF	Exp 1	21.36±0.15	31	<0.001	65
		Exp 2	22.69±0.62	33	<0.001	66
		Exp 3	23.08±0.11	34	<0.001	69
eat-2(ad1116)	Control	Exp 1	29.23±020	44	/	69
		Exp 2	29.56±0.35	45	/	61
		Exp 3	29.37±0.15	44	/	60
	Midlife Stage ADF	Exp 1	31.56±0.31	57	<0.001	66
		Exp 2	32.25±0.35	58	<0.001	68
		Exp 3	31.69±0.37	58	<0.001	65
daf-16(mu86)	Control	Exp 1	12.55±0.44	15	/	68
		Exp 2	13.18±0.05	15	/	63
		Exp 3	12.95±0.41	16	/	61
	Midlife Stage ADF	Exp 1	13.48±0.36	16	n.s.	69
		Exp 2	12.57±0.26	15	n.s.	68
		Exp 3	12.71±0.22	16	n.s.	63
skn-1(zu67)	Control	Exp 1	12.05±0.09	20	/	69
		Exp 2	12.21±0.74	20	/	64
		Exp 3	12.38±0.33	21	/	60
	Midlife Stage ADF	Exp 1	12.53±0.70	20	n.s.	62
		Exp 2	12.63±0.62	20	n.s.	69
		Exp 3	12.47±0.68	21	n.s.	66
daf-2(e1370)	Control	Exp 1	35.63±0.58	55	/	60
		Exp 2	36.18±0.55	55	/	65
		Exp 3	35.89±0.68	55	/	62
	Midlife Stage ADF	Exp 1	41.56±0.32	64	<0.001	60
		Exp 2	41.28±0.31	65	<0.001	65
		Exp 3	41.39±0.45	64	<0.001	61
hlh-30(tm1978)	Control	Exp 1	12.96±0.54	18	/	85
		Exp 2	13.12±0.27	18	/	69
		Exp 3	13.08±0.60	18	/	61
	Midlife Stage ADF	Exp 1	16.52±0.31	24	<0.001	68
		Exp 2	16.23±0.16	24	<0.001	62
		Exp 3	16.08±0.11	24	<0.001	69
N2	Midlife Stage ADF	Exp 1	21.36±0.15	31	/	65
		Exp 2	22.69±0.62	33	/	66

		Exp 3	23.08±0.11	34	/	69
hlh-30(tm1978)		Exp 1	16.52±0.31	24	<0.001	68
		Exp 2	16.23±0.16	24	<0.001	62
		Exp 3	16.08±0.11	24	<0.001	69
cpr-2(ok2833)		Exp 1	14.12±0.55	22	<0.001	69
		Exp 2	13.64±0.41	21	<0.001	64
		Exp 3	13.46±0.45	20	<0.001	60
cpr-5(ok2344)		Exp 1	17.96±0.46	25	<0.001	66
		Exp 2	17.16±0.53	25	<0.001	62
		Exp 3	18.29±0.57	26	<0.001	64
eat-2(ad1116)		Exp 1	31.56±0.31	57	/	66
		Exp 2	32.25±0.35	58	/	68
		Exp 3	31.69±0.37	58	/	65
eat-2(ad1116);cpr-2(ok2833)		Exp 1	20.36±0.28	34	<0.001	92
		Exp 2	19.85±0.06	34	<0.001	92
		Exp 3	19.36±0.17	34	<0.001	65
eat-2(ad1116);cpr-5(ok2344)		Exp 1	23.69±0.08	41	<0.001	67
		Exp 2	23.98±0.19	42	<0.001	84
		Exp 3	24.05±0.22	41	<0.001	62
daf-2(e1370)		Exp 1	41.56±0.32	64	/	60
		Exp 2	41.28±0.31	65	/	65
		Exp 3	41.39±0.45	64	/	61
daf-2(e1370);cpr-2(ok2833)		Exp 1	31.05±0.16	50	<0.001	88
		Exp 2	30.69±0.18	51	<0.001	82
		Exp 3	30.88±0.32	50	<0.001	65
daf-2(e1370);cpr-5(ok2344)		Exp 1	35.69±0.32	56	<0.001	62
		Exp 2	36.11±0.17	56	<0.001	65
		Exp 3	36.24±0.03	57	<0.001	63
daf-16(mu86)		Exp 1	13.48±0.36	16	/	69
		Exp 2	12.57±0.26	15	/	68
		Exp 3	12.71±0.22	16	/	63
daf-16(mu86);cpr-2(ok2833)		Exp 1	12.36±0.18	15	n.s.	77
		Exp 2	12.52±0.16	15	n.s.	71
		Exp 3	12.37±0.29	15	n.s.	82
daf-16(mu86);cpr-5(ok2344)		Exp 1	12.52±0.14	15	n.s.	90
		Exp 2	12.37±0.43	15	n.s.	66
		Exp 3	12.61±0.52	15	n.s.	65
skn-1(zu67)		Exp 1	12.53±0.70	20	/	62
		Exp 2	12.63±0.62	20	/	69
		Exp 3	12.47±0.68	21	/	66
skn-1(zu67);cpr-2(ok2833)		Exp 1	12.41±0.15	22	n.s.	92
		Exp 2	12.35±0.09	21	n.s.	66

		Exp 3	12.37±0.16	21	n.s.	68
skn-1(zu67);cpr-5(ok2344)		Exp 1	12.33±0.26	21	n.s.	64
		Exp 2	12.36±0.61	22	n.s.	74
		Exp 3	12.55±0.53	21	n.s.	101
N2	Control (L4440)	Exp 1	15.93±0.24	20	/	65
		Exp 2	16.14±0.15	21	/	62
		Exp 3	16.32±0.21	21	/	62
	Midlife Stage ADF	Exp 1	22.16±0.46	33	<0.001	63
		Exp 2	22.49±0.34	34	<0.001	60
		Exp 3	22.17±0.52	33	<0.001	78
let-363 RNAi	Control (L4440)	Exp 1	18.02±0.14	21	/	74
		Exp 2	18.11±0.33	21	/	105
		Exp 3	17.89±0.27	21	/	111
	Midlife Stage ADF	Exp 1	19.89±0.08	28	<0.001	68
		Exp 2	20.11±0.18	28	<0.001	69
		Exp 3	20.05±0.27	27	<0.001	63
lipl-3 RNAi	Control (L4440)	Exp 1	15.89±0.07	19	/	74
		Exp 2	16.23±0.37	19	/	89
		Exp 3	16.18±0.15	19	/	65
	Midlife Stage ADF	Exp 1	18.06±0.25	26	<0.001	60
		Exp 2	17.92±0.22	26	<0.001	67
		Exp 3	18.11±0.39	26	<0.001	61
N2	Midlife Stage ADF	Exp 1	22.16±0.46	33	/	63
		Exp 2	22.49±0.34	34	/	60
		Exp 3	22.17±0.52	33	/	78
let-363 RNAi		Exp 1	19.89±0.08	28	<0.001	68
		Exp 2	20.11±0.18	28	<0.001	69
		Exp 3	20.05±0.27	27	<0.001	63
lipl-3 RNAi		Exp 1	18.06±0.25	26	<0.001	60
		Exp 2	17.92±0.22	26	<0.001	67
		Exp 3	18.11±0.39	26	<0.001	61
**Plant-based peptone**						
Strains	treatment	group	Mean life Span±SEM(Days)	Maximum life span(Days)	P value	N
N2	Control	Exp 1	17.83±0.42	25	/	66
		Exp 2	18.20±0.15	25	/	78
		Exp 3	18.53±0.62	25	/	63
	Midlife Stage ADF	Exp 1	17.29±0.30	25	n.s.	61
		Exp 2	17.69±0.43	25	n.s.	65
		Exp 3	17.96±0.45	24	n.s.	73
eat-2(ad1116)	Control	Exp 1	30.11±0.01	46	/	60
		Exp 2	30.15±0.50	45	/	62

		Exp 3	30.41±0.61	46	/	63
	Midlife Stage ADF	Exp 1	30.56±0.55	45	n.s.	66
		Exp 2	30.21±0.31	45	n.s.	61
		Exp 3	30.25±0.28	45	n.s.	65
daf-16(mu86)	Control	Exp 1	13.29±0.18	16	/	62
		Exp 2	13.36±0.08	16	/	70
		Exp 3	13.51±0.19	17	/	63
	Midlife Stage ADF	Exp 1	13.15±0.77	16	n.s.	63
		Exp 2	13.41±0.69	16	n.s.	64
		Exp 3	13.93±0.82	17	n.s.	67
skn-1(zu67)	Control	Exp 1	12.98±0.09	21	/	71
		Exp 2	12.68±0.17	21	/	60
		Exp 3	12.66±0.63	21	/	60
	Midlife Stage ADF	Exp 1	12.75±0.18	20	n.s.	62
		Exp 2	12.85±0.52	20	n.s.	68
		Exp 3	12.83±0.07	21	n.s.	75
daf-2(e1370)	Control	Exp 1	36.55±0.11	57	/	70
		Exp 2	37.04±0.26	57	/	73
		Exp 3	36.61±0.38	56	/	62
	Midlife Stage ADF	Exp 1	37.26±0.16	56	n.s.	66
		Exp 2	36.64±0.05	56	n.s.	71
		Exp 3	36.83±0.18	57	n.s.	69

Control for RNAi: Feeding RNAi control clones with empty vector L4440. Mean lifespans were calculated by using Kaplan-Meier method; P values were determined using the log-rank test; SEM: standard error of the mean; N: number of worms used. n.s.: no significant difference.
